# Bacterial Succession and Community Dynamics of the Emerging Leaf Phyllosphere in Spring

**DOI:** 10.1128/spectrum.02420-21

**Published:** 2022-03-02

**Authors:** Wenke Smets, Lucia Maria Spada, Isabella Gandolfi, Karen Wuyts, Marie Legein, Babette Muyshondt, Roeland Samson, Andrea Franzetti, Sarah Lebeer

**Affiliations:** a Department of Plant and Microbial Biology, University of California, Berkeley, Berkeley, California, USA; b Department of Bioscience Engineering, University of Antwerp, Antwerp, Belgium; c Department of Earth and Environmental Sciences, University of Milano-Bicocca, Milan, Italy; Tufts University

**Keywords:** DNA sequencing, leaf colonization, microbial ecology, phylloplane-inhabiting microbes

## Abstract

Every year, deciduous trees shed their leaves, and when new leaves emerge next spring, they establish a characteristic bacterial leaf community. In this exploratory study, we assessed the bacterial phyllosphere (aboveground plant surfaces) of eight London plane trees (*Platanus × acerifolia*) in Antwerp and Milan by sampling weekly during leaf emergence and expansion. We sampled the surfaces of different tree compartments: leaves, leaf buds, branches, and trunk, for up to 6 weeks. Phyllosphere community composition was most strongly determined by tree compartment. Only the communities on the emerging leaves showed changing dynamics over time. The rate of change in the leaf phyllosphere composition, expressed as the beta dissimilarity between consecutive time points, was very high following leaf emergence, with decreasing speed over time, indicating that these communities stabilize over time. We also identified cooccurring groups of bacteria associated with potential stages of ecological succession on the leaves and accordingly named them general cluster, early cluster, middle cluster, and late cluster. Taxa of the general cluster were not only more abundant than the others on leaves, but they were also widespread on other tree compartments. The late cluster was most pronounced in trees surrounded by trafficked urban land use. This study mainly generates hypotheses on the ecological succession on the emerging leaves of deciduous trees in urban environments and contributes to understanding the development of the tree leaf phyllosphere in spring.

**IMPORTANCE** Improving our understanding of phyllosphere ecology is key in successfully applying bacterial biological agents or modulating the leaf microbiome in order to achieve valuable ecosystem services, such as plant protection, plant growth, air purification, and developing a healthy human immune system. Modulation of the phyllosphere microbiome in the field works only with variable success. To improve the impact of our applications in the field, a better understanding of the ecological principles governing phyllosphere dynamics is required. This exploratory study demonstrates how the combination of different analyses of a chronosequence of bacterial communities can provide new ecological insights. With a limited number of sampled trees, we demonstrated different indications of ecological succession of bacterial communities in the leaves and observed a potential impact of intensely trafficked land use becoming apparent in the leaf bacterial communities approximately 3 weeks after leaf emergence, consisting of a separate stage in community development.

## INTRODUCTION

Using plant-associated microbes in the phyllosphere, the habitat at the interface of plant and atmosphere, holds great potential for sustaining plant health, improving ecosystem productivity, air purification, and developing a healthy human immune system (1–5). However, without sufficient insight into the ecology of the phyllosphere, microbial treatments can unpredictably fail or might have unknown adverse effects, such as initiating phyllosphere dysbiosis and threatening plant health (6, 7). The emergence of new leaves on urban trees in spring provides a useful model for studying the dynamics of bacterial community assembly during leaf maturation in an outdoor setting.

In general, when a new habitat comes into existence (e.g., new leaf surface), we expect ecological succession to take place, where the colonization of the first pioneer organisms will be followed by different intermediate and climax species as the habitat changes over time. This is indeed what is observed when studying temporal patterns in the bacterial communities of apple blossoms. Shade and colleagues (8) found that persistent taxa present on apple blossoms clustered into six groups (pioneer, early, middle, late, climax, and generalists) and that the relative abundance of each group gradually changed over time. Previous studies on the leaf phyllosphere have observed the leaf communities to be dynamic as well, with diversity and composition changes over time depending on the location, season, and time frame of the study (9–13). However, ecological successional groups in the leaf phyllosphere have—to the best of our knowledge—not yet been identified.

Throughout the process of leaf phyllosphere development, a combination of selection, drift, dispersal, and evolutionary diversification appears to determine the community composition (14, 15). A greenhouse experiment with *Arabidopsis* plants by Maignien and colleagues (16) suggested that the development of phyllosphere bacterial communities on sterile plants is initially determined by the colonists that arrived first in the new habitat, by dispersal. The conditions, both environmental and those determined by the host plant, then exert a selective pressure on the bacteria present in the leaf phyllosphere (16–20). Therefore, in the greenhouse experiment of Maignien and colleagues (16), the development of the bacterial phyllosphere community was observed to be deterministic but with at least two different outcomes: with a prominent role of Pseudomonas in one set of plants and Acinetobacter in the other set of plants. The early colonizers seemed to determine which of the possible trajectories for community development would be followed (16). However, it remains unclear whether the same principles apply to phyllosphere communities in the field, where conditions and bacterial exposures may vary a lot more than in the greenhouse.

In less-controlled conditions, as plants germinate, bacteria on the original seed and in the germination environment, like the soil, appear to be the first phyllosphere colonizers (10, 21). In later stages of a plant’s life, the proportional importance of these initial bacterial sources for the phyllosphere community composition has been shown to decrease drastically, with more leaf-specific bacteria dominating the community, where “leaf-specific” is generally defined as not occurring in soil and repeatedly occurring on leaves (10, 14, 17, 21, 22). At any stage of host plant development, new bacteria can arrive on the phyllosphere through the air, through rainfall, and occasionally by vectors, like insects (23–25). These dispersal factors are not present or barely present in a greenhouse setting; hence field studies are required to gain more insight into how selection and dispersal relate throughout phyllosphere development. Additionally, we previously found that leaf phyllosphere community composition of trees surrounded by various proportions of different urban land uses were mainly affected by the area of green infrastructure versus the area of nongreen urban land use (referring to all urban land uses except green infrastructures) surrounding the tree (26). Hence, when studying phyllosphere development, it is important to take the environment of the plant host into consideration.

In this study, by examining the urban tree phyllosphere in spring, we aimed to explore the community temporal dynamics and differences between phyllosphere compartments in urban deciduous trees. We wanted to explore what insights we might gain from a fine-scale chronosequence with trees sampled every week. We included sampling of different phyllosphere compartments and focused our sampling efforts on eight London plane trees, a deciduous broadleaved tree species. Four of the trees were sampled three times in the week of leaf emergence and weekly throughout expansion in spring. We used *16S rRNA* gene amplicon sequencing to determine community composition.

## RESULTS

### General community composition.

After sequence processing, our data set consisted of 3,589,788 reads in a total of 171 samples and six blanks. The average number of reads per sample was 5,149 for leaf buds, 5,542 for leaves, 24,000 for branches, and 31,306 for trunks. This discrepancy was caused by the high number of chloroplast reads (which were removed during data processing) in bud and leaf samples. We found 4,873 different amplicon sequence variants (ASVs) in our samples, representing 1,009 genera, 387 families, and 34 phyla. Then, blank samples were discarded from the data set, and external contaminants were removed, discarding 16 ASVs representing approximately 0.06% of all reads. About 14% of all ASVs were shared between Antwerp and Milan. However, due to slightly different library preparation approaches in the two cities (reflected in different read length variations), this could be an underestimation of the amount of ASVs that were shared. We found that 39% of all genera were shared between the two cities.

**FIG 1:**
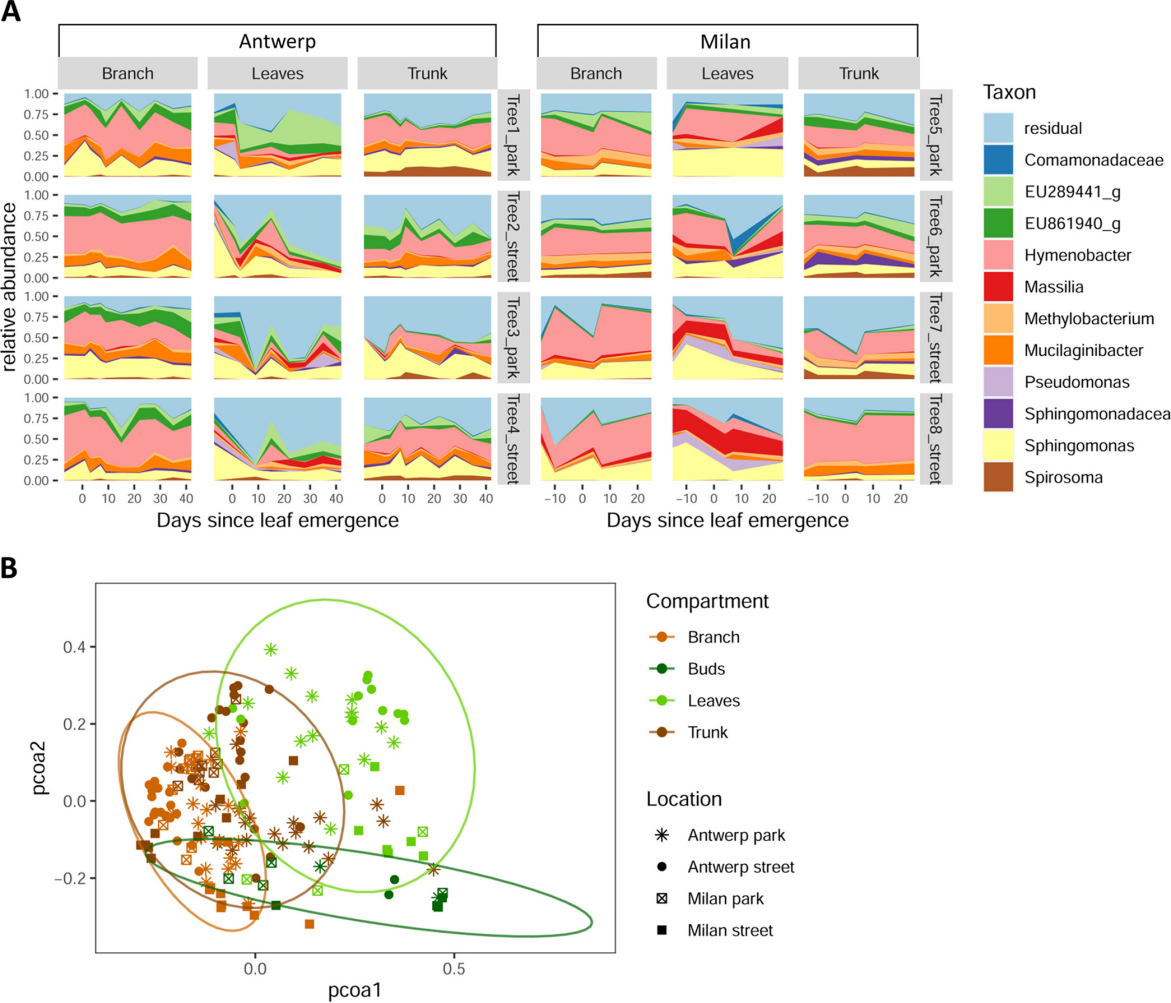
(A) Stacked area plots visualizing community composition of the 11 most abundant genera per tree over time (when a genus was unknown, it was named by its family name). The *x* axis represents time, the *y* axis represents relative abundances, the columns of plots represent the different compartments in each city, and the rows of plots represent different tree individuals. (B) Principal coordinates analysis (PCoA) plot showing the compositional variation of all phyllosphere samples of this study in a two-dimensional space. The colors indicate the compartments of the sample, and the shape of the symbol refers to the location where it was taken.

The most abundant genera in the data set were Hymenobacter (23.9%), Sphingomonas (17.1%), Mucilaginibacter (6.4%), *EU861940* of the family Acetobacteraceae (5.9%), and *EU289441* of the family Beijerinckiaceae (5.4%). Using a bar plot, we visualized their relative abundances over time (Fig. 1A). Not many indicator genera were found using the indicator analysis, but Hymenobacter and Mucilaginibacter were significant indicator genera of the trunk and branches, whereas Fimbriimonas was a significant indicator genus for the trunk alone.

Using permutational multivariate analysis of variance (PERMANOVA) on the whole data set at genus level, we found that phyllosphere compartment, city, land use (park or street), time since leaf emergence, and tree individual, as well as and several of their interactions, significantly affected the variation in community composition; however, the effects were to various degrees (Table 1). To confirm that these factors were not merely significant because they affected the size of community variation within a group rather than the average composition, permutational analyses for homogeneity of multivariate dispersions (PERMDISP2) analyses were done. PERMDISP2 confirmed homogeneity of dispersion among groups defined by tree compartment, city, land use, and tree individual (all *P* values were greater than 0.4), indicating that the factors identified by PERMANOVA are indeed important for community composition. Phyllosphere compartment was the most important factor for community composition, which is visualized in the principal coordinates analysis (PCoA) plot (Fig. 1B). The effect of city is confounded with different library preparations for samples in the two different cities; hence, we cannot determine the actual effect of city on the community except that up to 6% of community variation is potentially determined by city (Antwerp versus Milan) in this study.

**TABLE 1:** Contribution of tested factors to phyllosphere compositional variation^*a*^

Factor	*R* ^2^	*P* value
Phyllosphere compartment	0.24	<0.001
City	0.06	<0.001
Park/street	0.02	<0.001
Tree individual	0.01	0.005
Days since leaf emergence	0.007	0.046
Compartment: city	0.06	<0.001
Compartment: park/street	0.04	<0.001
Compartment: days since leaf emergence	0.02	0.006

a All factors tested in the general permutational multivariate analysis of variance (PERMANOVA model (10,000 permutations) significantly contribute to the phyllosphere compositional variation. Phyllosphere compartment refers to whether the sample was taken from the leaves, buds, branches, or trunk. Colons (:) indicate an interaction between factors (only the interactions with leaf compartment were tested). The *P* value column indicates that all tested factors were found to be significant, and the *R*^2^ column indicates the contribution of the respective factors to the community variation.

### Different phyllosphere compartments.

Because phyllosphere compartment showed the greatest effect on the phyllosphere community composition in our study, we further investigated how community structure differed between compartments. Core genera of the different phyllosphere compartments are indicated in Table 2. We found only one core genus in the leaf phyllosphere (Sphingomonas), but the community of this compartment was developing over the course of this study. At the beginning of the sampling period, the members of the leaf phyllosphere may not have reflected a representative leaf phyllosphere, and even at the end of the sampling period, 42 days after leaf emergence, we could not confirm that the leaf phyllosphere reached a mature stage, which is generally sampled in other phyllosphere studies.

**TABLE 2:**
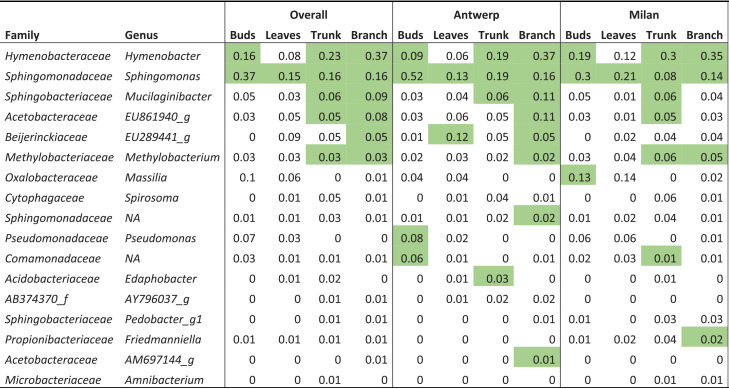
Mean relative abundances and taxonomy of the core genera of different phyllosphere compartments^*a*^

a The mean relative abundances and taxonomy of the core genera (which is defined as being present in more than 95% of the samples of the specified compartment) of the different phyllosphere compartments are shown. Sequences that could not be specified to genus level in the reference database are indicated with NA. Green shading indicates that the genus was found to be a core genus in the corresponding compartment. We also determined core genera of the compartments in the separate cities.

**FIG 2:**
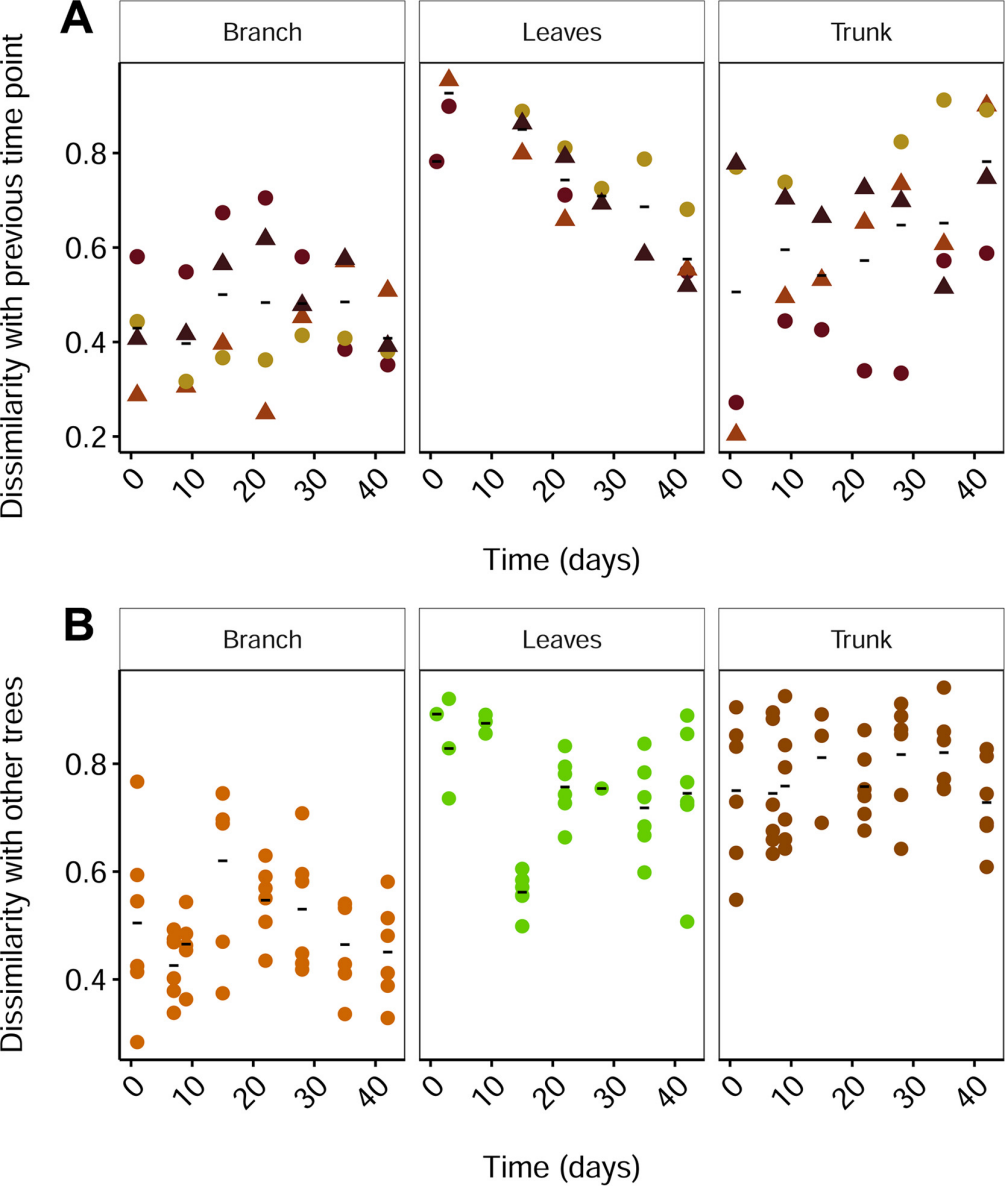
Community dynamics of Antwerp trees throughout time (number of days since leaf emergence) expressed as follows: (A) Dissimilarity between two consecutive time points, with each tree individual indicated by a different color. The triangles are street samples, and the circles are park samples. (B) Dissimilarity between trees within time points. The mean of each time point is indicated with a black horizontal line.

To study the community dynamics of the Antwerp trees, we used the Bray-Curtis dissimilarities of two consecutive time points of the same tree and phyllosphere compartment. Hence, a higher dissimilarity indicates a larger and therefore faster change in a community during 1 week, whereas stable dissimilarities over time indicate a stabilized community. The highest dissimilarities were observed for the emerging leaf phyllosphere samples, despite a shorter time between sampling points in this period (Fig. 2A). These results indicate that during the first weeks after leaf emergence, the leaf phyllosphere communities were changing at a higher rate than the communities on the branches or trunk. Furthermore, a significant negative Kendall rank correlation of the dissimilarities over time was observed in the emerging leaf communities (*P* < 0.001, τ = −0.69), which suggests that new leaf phyllosphere communities stabilize over time. A significant trend was absent in trunk (*P* = 0.103) or branch (*P* = 0.94) communities. The same conclusions hold true when analyzing the data set at the genus level, although the downward trend in the leaf compartment became less steep (τ = −0.52) than at the ASV level. In addition, we found that dissimilarities between different trees within one time point showed no significant trend over time and was generally lower in the branch communities (Fig. 2B). The lack of a decreasing trend for leaves indicates that the developing leaf phyllosphere communities did not evolve toward a similar community composition.

### Community development on new leaves.

**FIG 3:**
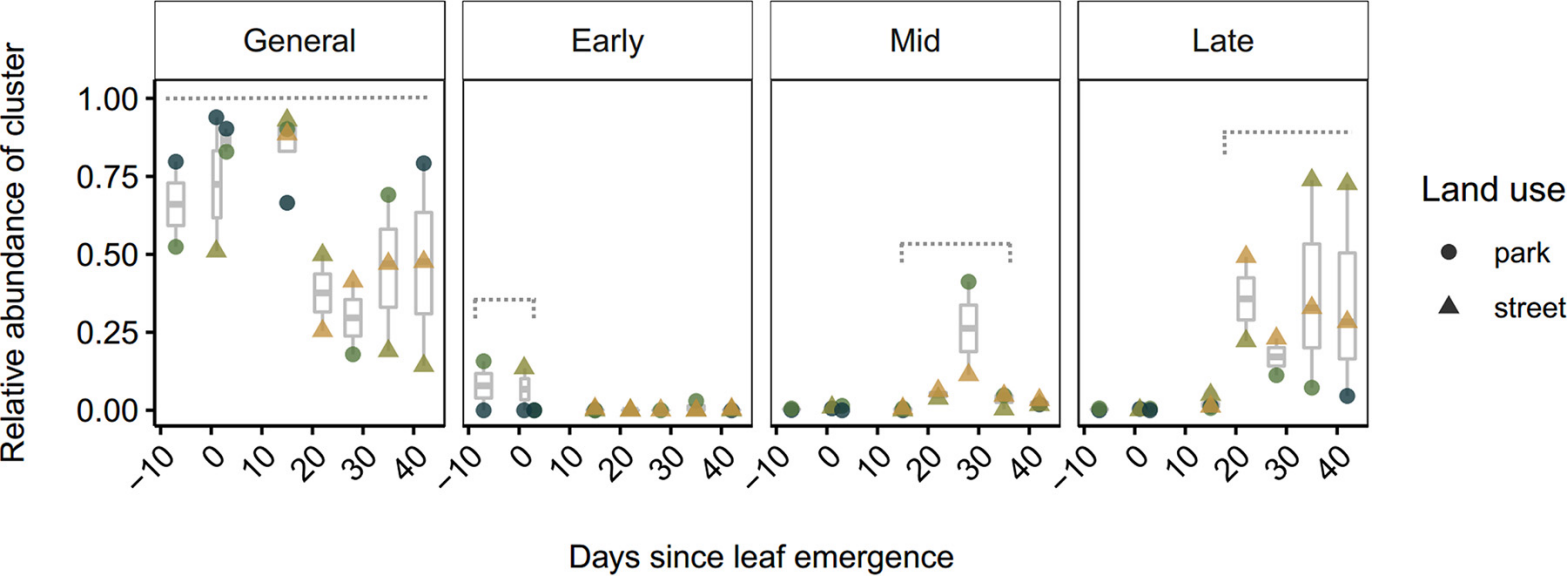
Relative abundances of different ASV clusters in the leaf phyllosphere of Antwerp over time. Each color represents a tree. Boxes with whiskers were generated per time point. The time frame of the respective clusters is indicated with gray dashed lines. The Milan data are compared with the Antwerp data in Fig. S4.

The more abundant Antwerp leaf ASVs were divided into four clusters based on their cooccurrences in the leaf samples. We found that cluster abundances showed temporal trends, indicating different stages of leaf phyllosphere succession. We arbitrarily named the clusters according to their relative importance in time: general, early, middle, and late (Fig. 3). Remarkably, the late cluster seemed more pronounced in the trees located next to busy streets compared to the park trees (Fig. 3). Furthermore, when using the ASV cooccurrence clusters generated for the Antwerp samples, the Milan leaf phyllosphere ASVs, although representing far less samples and possibly biased due to different library preparations, seemed to follow the same temporal patterns, except for the early cluster, which is completely absent in Milan, and potentially for the late cluster, which appeared to become more abundant at an earlier stage (Fig. S3). The most abundant ASVs representing each of the clusters are listed in Table 3. The general cluster seems to contain the most dominating taxa. An overview of all ASVs per cluster can be found in Table S4. When doing the same ASV cluster analysis for the trunk, no temporal patterns were found, as clusters were mainly associated with host tree individual (Fig. S4). When doing the same ASV cluster analysis for the branch samples, a gradual increase of one cluster throughout the study period was observed, but it did not represent multiple stages of succession (Fig. S5).

**TABLE 3:** Most abundant ASVs for each of the cooccurrence clusters, their statistical support, and average relative abundance in Antwerp leaf samples^*a*^

ASV	Support	Avg abun (%)
General cluster		
* Sphingomonas 1*	1	5.7
* EU289441_g 1*	0.992	4.6
* EU289441_g 2*	0.953	4.6
* Mucilaginibacter 1*	0.978	3.9
* EU861940_g 1*	0.977	3.7
* Sphingomonas 2*	0.998	3.5
* Beijerinckiaceae 1*	0.956	2.7
* Hymenobacter 1*	0.972	1.4
* Pseudomonas 1*	0.974	1.1
* Hymenobacter 15*	0.972	1
Early cluster		
* Hymenobacter 29*	0.491	0.39
* Sphingomonas 10*	0.487	0.2
* Chthoniobacteraceae 3*	0.361	0.14
* EU289441_g 8*	0.347	0.14
* Sphingomonas 7*	0.267	0.13
* EU289441_g 7*	0.208	0.13
* Hymenobacter 64*	0.056	0.1
* Beijerinckiaceae 3*	0.056	0.1
* Edaphobacter 2*	0.046	0.1
* Planctomycetaceae 2*	0.002	0.08
Middle cluster		
* Chamaesiphon 1*	0.916	0.64
* Sphingomonas 16*	0.916	0.51
* Paracoccus 2*	0.82	0.42
* Cronobacter 1*	0.916	0.32
* Thermomonas*	0.897	0.31
* Intrasporangiaceae 1*	0.916	0.28
* Qipengyuania*	0.916	0.27
* Pseudarthrobacter*	0.785	0.24
* Bacillus 3*	0.536	0.23
* Acetobacteraceae 3*	0.913	0.22
Late cluster		
* Massilia 2*	0.59	2.1
* Bacillus 1*	0.772	1.07
* Erwinia 2*	0.395	1.05
* Skermanella 1*	0.772	0.86
* Pantoea*	0.793	0.85
* Rubellimicrobium 1*	0.769	0.8
* Clostridium 1*	0.592	0.77
* Noviherbaspirillum*	0.63	0.74
* ALVU_g 1*	0.772	0.63
* Blastococcus 1*	0.793	0.54

a The table shows the 10 most abundant amplicon sequence variants (ASVs) for each of the cooccurrence clusters with their respective support for that cluster and average relative abundance in the Antwerp leaf samples. ASVs are named after their genus (or their family when the genus was unknown) and their order of abundance within this genus. The clusters with all of their corresponding ASVs can be found in Table S4.

### Generalism in the phyllosphere.

**FIG 4:**
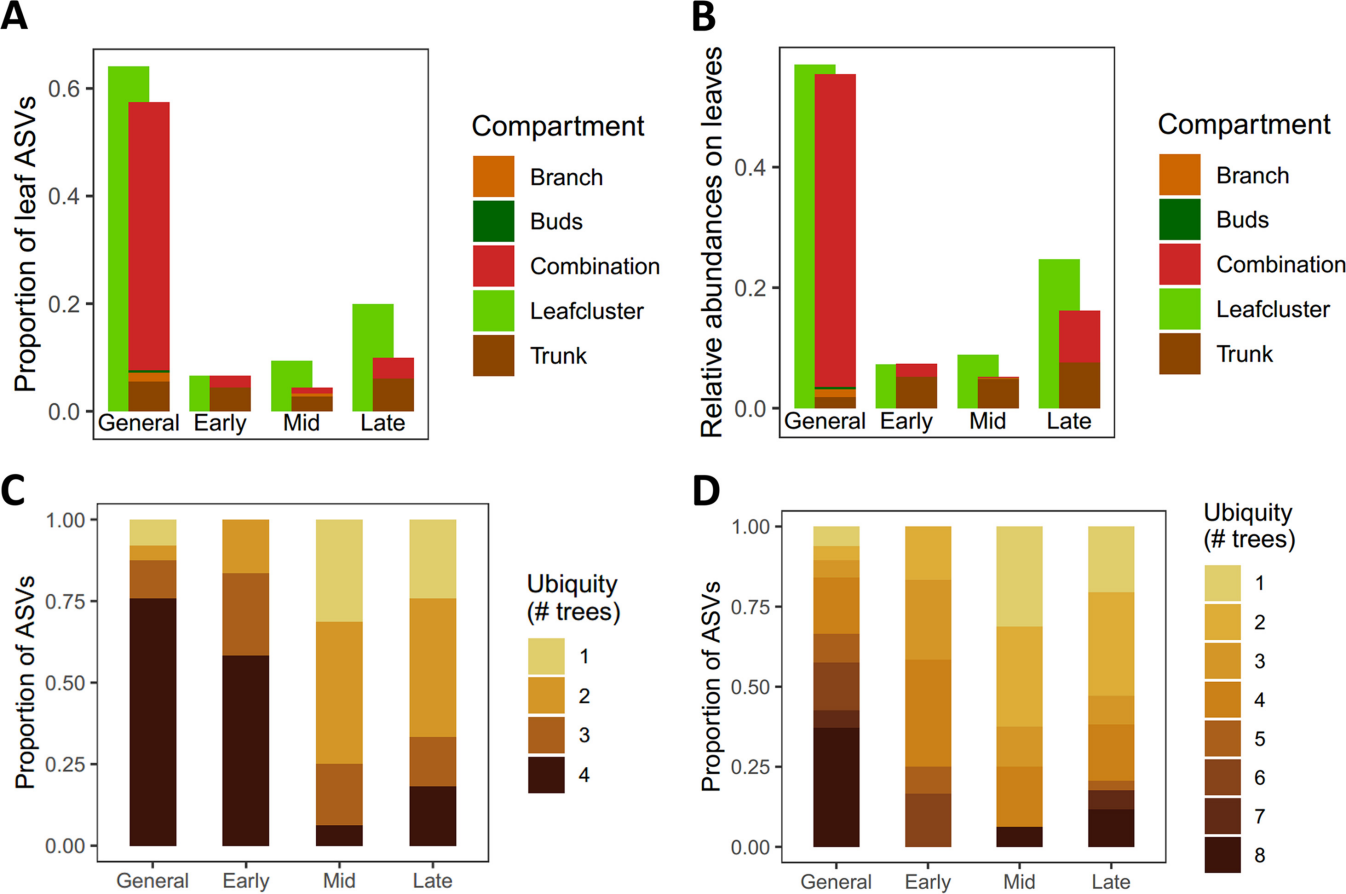
Amplicon sequence variant (ASV) overlap between phyllosphere compartments and ubiquity across trees. The analyses were done per leaf cluster, which is indicated in the *x* axis. The top graphs (A, B) show the contributions of leaf cluster ASVs to the leaf community (green bars) and to what extent the ASVs in these leaf clusters overlap with other compartments (colored bars). The green left-hand bars show the average proportion of ASVs (A) and relative abundance (B) of the leaf cluster in the leaf community during the relevant time frame of the cluster. The right-hand colored bars overlapping with the green bars represent the ASVs found in both the respective leaf cluster and in at least one of the other tree compartments. The colors in the right-hand bars indicate which other compartment the leaf cluster ASVs were found in. If the leaf ASVs were found in more than one other compartment, they were categorized as “combination.” The part of the green bar that has no overlap with the other bars therefore represents ASVs that were found only in the leaf compartment. Per cluster, ASV ubiquity in trees (C, D) was also visualized as the number of trees in which an ASV was present, with a high ubiquity corresponding to a presence in all four trees in Antwerp (C) or all eight trees in both cities (D).

To estimate the extent to which the leaf ASVs are adapted to different phyllosphere compartments, we calculated what proportion of ASV overlap existed between the leaf compartment and the other compartments. For each leaf cluster, we identified which ASVs were also found in at least one of the other compartments and how much these ASVs contributed to the leaf community (Fig. 4A and B). The general cluster and early cluster of the leaves showed a large overlap with the other tree phyllosphere compartments (more than 90%; ratio of colored bar and green bar in Fig. 4A and B), whereas the middle cluster and late cluster showed a smaller overlap with the other compartments (47 to 65%). In addition, the general cluster ASVs showed greater ubiquity across the different trees than the middle and late clusters (Fig. 4C and D).

## DISCUSSION

### Differences between phyllosphere compartments.

We found that phyllosphere compartment (trunk, branch, leaf buds, or leaves) of the urban London plane trees was the most important factor in explaining community variation in our study (Table 1; Fig. 2). This means there were larger community variations between phyllosphere compartments within one tree than between different trees (same compartment) over the eight trees we sampled occurring in the different urban contexts of Belgium and Italy. Despite tree compartment being the most important factor in our study, many of the core genera were shared across phyllosphere compartments (Table 2). As we sampled only four trees per city, we may be overestimating the amount of core taxa defined per city and compartment. Nevertheless, our findings are in accordance with previous studies that included multiple plant compartments, as they also observed many shared bacterial taxa despite the consistent observations of different phyllosphere compartments harboring very distinct community compositions (27–30).

Additionally, we found indications that community dynamics differed between the different compartments over the sampling period. On the one hand, the change of communities on the branches and trunks was stable over time, with the branches showing smaller changes between weeks than the trunks (Fig. 2A). On the other hand, we observed indications for specific community dynamics and ecological succession in the emerging tree leaf phyllosphere in spring, which we did not in the branches or trunk. This outcome is to be expected because the time frame of this study was targeting leaf development.

### Leaf phyllosphere development.

By using community dissimilarity between consecutive time points, we visualized the community change rate over time (Fig. 2A). For the four trees in Belgium, we observed that the leaf community change rate is high in the beginning as the first colonizers arrive and the community starts to form and then gradually decreases as the community develops further. For the four trees in Italy, this was difficult to determine as the time points were not at regular intervals. Our observations for Belgium are in line with the observations of Maignien and colleagues (16), in which greenhouse phyllosphere communities showed decreasing changes over a similar time span. The downward trend of change rate in the leaves indicates that the leaf communities change less and therefore start stabilizing as they move closer to an implicit final composition. However, the duration of our study was limited to 6 weeks after leaf emergence and did not cover the entire growing season. As a consequence, the results (Fig. 2A) did not allow us to determine what degree of stability (if any) the leaf phyllosphere community can reach over time because the downward trend of dissimilarities is still constant at the end of this study. Of note, our study time frame is also focused on bacterial community development on the leaves, which did not allow us to compare the development on the leaves with that on the woody tissues. With only four trees included in this chronosequence, we nevertheless detect differences in both community structure and dynamics between different tree compartments in spring.

As the leaf communities seem to progress toward a final implicit composition in Fig. 2A, we expected the stabilizing processes in the leaf phyllosphere (e.g., by leaf-specific conditions) would drive leaf communities of different trees toward a common final composition. To assess whether we could detect this, we compared leaf communities between trees within time points (Fig. 2B). If there would indeed be a common final composition, we would expect their dissimilarities to decrease over time, because the communities would become more similar as they approach a common final composition. The dissimilarities between trees, however, showed no change over time but rather a constant dissimilarity, which led to the hypothesis that the leaf phyllosphere development is a process that unfolds differently in each host tree individual (and/or in its location) and will likely result in different community compositions if the communities indeed reach a more-or-less stable, final composition. Both a longer period of time and more trees need to be covered in future research to study these hypotheses.

Based on ASV cooccurrences, we distinguished four clusters of ASVs, which showed temporal patterns and potentially represent ecological succession in the leaf phyllosphere. The clusters were named according to the time frame in which they exhibited higher relative abundances: early cluster, middle cluster, late cluster, and general cluster; with the latter usually representing more than 40% of the abundance in the community. Although this cluster analysis is based on data from only two trees in each of the land uses, the late cluster was remarkable for its high relative abundances in trees in a trafficked urban setting, compared to trees in the park. The early cluster occurred only at low abundances and generally showed weak support, which would be a logical consequence of the initial colonization being rather stochastic and dependent on which ASVs arrive first (16).

On the one hand, we observed in a previous study that the shift in bacterial leaf phyllosphere communities typical for nongreen urban land use compared to green urban land use was at least partially dispersal-limited (26). On the other hand, in this study, we observed a large, dominant group of ASVs that seem to be present in four trees spread out over a city and therefore do not seem to be dispersal-limited. In our current study, the general leaf cluster shared many ASVs with the other parts of the tree, potentially indicating that a large proportion of ASVs in the leaf phyllosphere are phyllosphere generalists through successional time as well as throughout different phyllosphere compartments (Fig. 4A and B). These findings are similar to those of Massoni and colleagues (28), who observed the phyllosphere of several herbaceous species to be dominated by bacteria with a phyllosphere generalist lifestyle based on their occurrence on different host plants in both flowers and leaves. In addition, most ASVs of the generalist cluster were present in trees far apart in the city of Antwerp or even in both Antwerp and Milan, cities 900 km apart. The library preparation of the samples taken in Antwerp and Milan is different; hence, direct comparisons of these data sets, especially at ASV level, is likely biased. However, this makes it even more remarkable that some of the exact same ASVs of the general cluster in Antwerp are also found in Milan. Due to the different library preparations, the overlap between both cities in this study is likely an underestimation, and we limited our conclusions to trends detected within each data set separately (unless stated otherwise). The observation of this general, abundant, seemingly ubiquitous, cluster in our study indicates that, in addition to dispersal limitation detected by Wuyts and colleagues (26), a group of dominant, ubiquitous phyllosphere generalists determines a large portion of community structure.

### Impact of nongreen urban land use.

Leaf phyllosphere communities of different host plant species were previously shown to be affected by an urban gradient, and, more specifically, by land use and air pollution (26, 31, 32). In this study, with the rise of the late cluster, between 2 and 3 weeks after leaf emergence in Antwerp, we detected a potential difference between trafficked urban land use and park. The value of this observation is limited due to having only two trees for each land use. However, when analyzing beta dissimilarities of all eight trees using PERMANOVA, we again detect a significant effect of land use (Table 1). This analysis, however, includes data from data sets that were generated using different library preps, which is known to cause biases. Hence, on the one hand, this analysis was done at the genus level, which should decrease smaller nucleotide-scale biases; on the other hand, the remaining bias would already be covered by the “city” effect in the PERMANOVA model. Although, again, eight trees are not a large amount, the land use effect we detect might have some value. In a previous study, we found that for the London plane tree leaf communities, a decreasing amount of surrounding green infrastructure in an urban landscape may decrease the relative abundances of other dominant typical leaf-associated bacteria such as Sphingomonas, Hymenobacter, and Beijerinckiaceae (generalist cluster in our present study) by increasing limited dispersal between plants and may increase the abundances of other bacteria from urban sources, such as Skermanella and Chroococcidiopsis (late cluster in our present study) (26). Furthermore, Wuyts and colleagues (26) showed that in addition to the effect of the surrounding green infrastructure, air pollution was linked to the extinction and introduction of several species. Besides the surrounding green infrastructure and air pollution, the slight temperature increases of a built-up area compared to a park area could also have a selective effect. We observed that the late cluster was more pronounced in the trees located next to busy streets, which experience slightly higher ambient temperatures than parks (33). Additionally, the late cluster appeared earlier in the trees in Italy (Fig. S3), which generally has a warmer climate than Belgium. These results are in agreement with a study by Aydogan and colleagues (34), who observed a decrease of Sphingomonas, Hymenobacter, and Methylobacterium (general cluster in this study) and an increase of Erwinia, Pantoea, Clostridium, and Bacillus (late cluster in this study) in grassland phyllosphere under elevated temperatures compared to unaltered temperatures. The observations in this study are based on a limited number of trees, but the community shift to higher abundances of species that occur in the late cluster of this study can be recognized in several other leaf phyllosphere studies.

This article reports on an exploratory study focusing on an observational chronosequence of bacterial phyllosphere communities on different tree compartments in spring. We observed a large overlap of ASVs between the different phyllosphere compartments (leaves, branches, and trunk) despite the very distinct compositions and dynamics in the different compartments. We also explored temporal dynamics of the phyllosphere communities, but because these dynamics analyses included only a limited number of trees, many of the generated hypotheses remain to be tested in future research. The frequent and consistent temporal sampling, however, led us to explore unique avenues of phyllosphere ecology. Using community dissimilarities between consecutive time points, we visualized community dynamics indicating decreasing change of the bacterial community composition on the emerging tree leaves over time. Our observations hint that this leaf community development follows a unique trajectory within each plant individual. Nevertheless, overall, we managed to detect ecological succession in the emerging tree leaves in spring and were able to identify four groups of leaf bacteria (clusters) exhibiting different temporal patterns during this process. Leaf community dynamics furthermore indicated that community development was still continuing at the end of this study, 6 weeks after leaf emergence. In this study, a large part of the emerging tree leaf phyllosphere consisted of phyllosphere generalists, present in different phyllosphere compartments, on different trees, and throughout leaf phyllosphere development. This indicates that the main structure of different phyllosphere communities of the urban London plane trees is determined by mostly ubiquitous ASVs. Nevertheless, distinct differences between communities can still be detected due to variation of relative abundances and less ubiquitous ASVs. More research endeavors in the future focusing on regular temporal sampling hold much promise for better understanding phyllosphere communities.

## MATERIALS AND METHODS

### Sampling.

Phyllosphere samples, including the trunks, leaf buds, branches, and leaves, of eight selected urban London plane trees (*Platanus × acerifolia*) were collected weekly throughout the spring season, from 29 March 2018 to 29 May 2018 (Table S1), resulting in 180 samples. This time frame was chosen to cover phyllosphere samples ranging from just before to well after leaf emergence. Leaf emergence of the London plane trees occurred around 11 April 2018 in Milan and around 17 April 2018 in Antwerp. The London plane tree was selected as host species because it is a common tree throughout Europe, often used in urban greening, it is easy to recognize, and its canopy is usually out of reach for people to touch, which decreases the chance of human contamination. In order to take into account different urban settings, four trees were selected in Antwerp, Belgium, and another four were selected in Milan, Italy. Antwerp experiences a maritime temperate climate, with lowest and highest mean air temperatures of 3°C in January and 19°C in July, respectively, and a mean annual rainfall of 848 mm (1981 to 2010; Royal Meteorological Institute of Belgium [RMI], http://www.kmi.be). Antwerp has approximately half a million inhabitants and covers about 200 km^2^. Milan experiences a warm temperate climate, with lowest and highest mean air temperatures of 2.5°C in January and 24°C in July, respectively, and a mean annual rainfall of 920 mm (1971 to 2000; Meteorological Service of the Italian Air Force). Milan has approximately 1.4 million inhabitants and covers about 180 km^2^. The surrounding urban land use of the sampled trees is illustrated in the maps in Fig. S1, and the geographic coordinates of the sampled trees can be found in Table S2.

On each sampling day, all four trees within one city were sampled. Sampling days were not the same in Antwerp and Milan as we expected leaves to emerge earlier in the warmer climate of Milan than in Antwerp. In addition, the sampling intensity in both cities differed, with 10 time points in Antwerp and five time points in Milan. From each tree, one smaller branch (no larger than 3 cm diameter) was cut from the lower canopy (2.5 to 8 m above the ground) using a telescopic pruner. The branches were intercepted with gloves (decontaminated with 70% ethanol) before touching the ground. From each branch, immediately, five to eight leaf buds or, after leaf emergence, two to six undamaged leaves were cut from the branch with scissors using gloves sterilized on site with 70% ethanol. The buds or leaves of each tree were put in sterile 50-mL tubes (Greiner Bio-One) and kept on ice for transportation to the lab. The branches were sampled using a sterile swab (Copan FLOQSwab; MLS) dipped in sterile leaf wash buffer as prepared by Redford and Fierer (9). The swab was then used to thoroughly swab bacteria off the branch surface of a 20- to 40-cm branch length. To sample the trunks, an area of 100 cm^2^, between 70 and 210 cm above ground level, was swabbed in the same way. We avoided swabbing the same trunk surface more than once during consecutive weeks and randomly changed the cardinal direction of the sampling. The swabs of branch and trunk were kept in Eppendorf tubes with 1 mL leaf wash buffer on ice for transportation to the lab. In the lab (at most 4 h after sampling), 5 mL of leaf wash buffer was added to the 50-mL tubes with leaves or buds. To suspend the phyllosphere bacteria, 50-mL tubes with leaves or buds and Eppendorf tubes with swabs were vortexed for 5 min at maximum speed with the Vortex Genie 2 (MoBio). The tubes with leaves were centrifuged at 1,000 × *g* for several seconds to spin down most of the buffer still sticking to the leaves. Leaves and swabs were removed from the tubes, and the remaining wash solution was centrifuged in aliquots of 2 mL at 12,000 × *g* for 2 min, and the supernatant was discarded. The final pellet was resuspended in 750 μL of bead solution (QIAamp PowerFecal DNA kit, Qiagen). This suspension was stored at −20°C until further processing.

### DNA sequencing.

The samples of the different cities were processed locally, in two different labs. DNA extraction of the samples was done with the QIAamp PowerFecal DNA kit (same buffers and procedure as QIAamp PowerSoil DNA isolation kit), according to the manufacturer’s instructions, except for the final elution step, which was done with only 60 μL elution buffer for increased DNA concentration. For each DNA extraction kit, at least one kit blank was included (extraction without sample). In both the Milan and Antwerp labs, the DNA extraction protocol and the hypervariable V4 region of the *16S rRNA* gene (primers 515F/806R as described by Caporaso et al. [35]) to be amplified were the same; however, the DNA preparation protocol for Illumina sequencing slightly deviated between the labs, as described below.

In Antwerp, PCRs were performed with barcoded primers (IDT) as described by Kozich and colleagues (36). For each sample, a 20-μL reaction mixture was prepared with 5× Phusion HF buffer, DMSO 3%, dNTPs 0.2 mM each, 0.5 μM each primer, and 0.02 U/μL Phusion high-fidelity DNA polymerase (ThermoFisher Scientific). Cycling conditions were: initial denaturation at 95°C for 2 min; 25 cycles of 95°C for 20 s, 55°C for 15 s, and 72°C for 1 min; and a final extension at 72°C for 10 min. PCR blanks were included. Amplicons were purified using magnetic beads of Agencourt AMPure XP (Beckman Coulter) according to the manufacturer’s instructions. The DNA concentration of purified samples was measured using a Qubit 3.0 fluorometer followed by equimolar pooling of all samples and blanks into a “library.” The library was loaded onto a 0.8% (mass/vol) agarose gel, which was run at 60 V for 50 min. Bands of approximately 380 bp were cut out and extracted with the Nucleospin gel and a PCR clean-up kit (Macherey-Nagel). The final library was diluted to 2 nM, based on the DNA concentration determined by the Qubit 3.0 fluorometer. The library was sequenced at the Centre for Medical Genetics (Edegem, Belgium) with Illumina MiSeq, using the 500-cycle MiSeq reagent kit v2 (Illumina).

In Milan, PCRs were performed with the same primers, except that, at the 5′ end of each primer, an in-house-designed 6-bp barcode was included. For each sample, two 60-μL reaction mixtures were prepared as in Antwerp and run under the same cycling conditions. Amplicons were purified with the Wizard SV gel and PCR clean-up system (Promega Corporation, Madison, WI), and purified DNA was quantified using Qubit (Life Technologies, Carlsbad, CA). Groups of nine amplicons bearing different barcode pairs were pooled to build a single library. Further library preparation with the addition of standard Nextera indexes (Illumina, Inc., San Diego, CA) and sequencing with Illumina Miseq (Illumina, Inc., San Diego, CA) using the 600-cycle MiSeq reagent kit v3 were carried out at the Consorzio per il Centro di Biomedicina Molecolare (CBM, Trieste, Italy). The sequencing data of this study were made available under study accession number PRJEB39205 in the European Nucleotide Archive.

### Data processing.

The raw sequencing data were processed using the DADA2 package (37) in the R environment (38). In short, sequence reads with at least one ambiguous base, reads containing the lowest possible quality score of 2, and reads with more than two “expected errors” were discarded. The reads of the sequencing run in Antwerp were of sufficient quality to truncate at position 251. For the Milan data processing, forward reads were truncated at position 210, whereas the reverse reads were truncated at position 170. These positions were determined based on the overall drop of quality score of the base pairs after these positions. Next, sample inference (DADA2’s core algorithm) was performed, followed by read merging. A sequence table containing all amplicon sequence variants (ASVs) was constructed, chimeras were removed, and taxonomy was assigned using the EzBioCloud 16S rRNA gene database, downloaded on 1 August 2018. The data were processed further using the tidyverse set of packages (39) and the in-house-developed “tidyamplicons” package (github.com/SWittouck/tidyamplicons). Nonbacterial reads (i.e., chloroplasts and Archaea) were discarded. For the samples taken in Antwerp, reads with less than 251 bp and more than 256 bp were discarded. For the samples taken in Milan, read length varied more (probably due to slightly different methodology) and reads with less than 234 bp and more than 265 bp were discarded. These slight differences between Antwerp and Milan may interfere when comparing data at the ASV level. As we expect the results to be more robust at a higher taxonomic level, we decided to use the data at the genus level for analyses whenever considering the whole data set. Whenever doing analyses at the ASV level, they were done for only one city or for each city separately. No normalization or subsampling of reads was done (unless specified otherwise for selected analyses), but samples with less than 200 reads were discarded (losing mainly leaf samples: 38 leaf samples out of 48 were retained).

Statistical analyses were also done in the R environment. Graphs were generated using the ggplot2 package (40). Whenever the *P* value was under 0.05, the result was considered significant. Bray-Curtis dissimilarities were calculated upon which permutational multivariate analysis of variance (PERMANOVA) was performed, and the levels of dispersion between groups of each factor were compared (PERMDISP2) using the vegan package (41). The R code used for the analyses is available in the supplemental material.

### Determining contaminants.

To identify contaminating ASVs, we used the compositions of the six kit blanks. We considered two types of contamination: external contaminants and cross-contaminants. We decided to make the distinction for each ASV occurring in both blanks and samples (Table S3) based on several considerations: abundant taxa in the phyllosphere samples or human microbiome samples in the same run could have leaked to the blanks and therefore potential phyllosphere ASVs were considered cross-contamination, whereas the human-associated ASVs were considered external contamination (human-associated samples were sequenced in the same run); additionally, some taxa can be recognized as typical kit contaminants and were considered external contamination (42). After careful taxon-by-taxon consideration, we discarded the external contaminants from our data set (discarding approximately 0.06% of all reads). In addition, we studied distributions of external contaminants in the samples to determine a read-number threshold for ASVs to be considered present in a sample to determine a threshold that would get rid of roughly 50% of the contaminants (supplementary R code).

### Compartment-specific taxa.

We determined indicator taxa to examine which taxa were associated significantly more with one or two phyllosphere compartments compared to the other compartments. Indicator taxa are biological indicators of groups of sites representing habitat types, or in this case, the phyllosphere compartments. This means that the abundances of the indicator taxa can help in predicting the phyllosphere compartment to which the community belongs. This analysis was limited to samples that were taken at the final time point to exclude dependency between samples. Abundances were centered-log-ratio-transformed, and the R package indicspecies (43) was used to determine the indicator taxa (at genus level) for each phyllosphere compartment.

To determine taxa which were strongly associated with one or more of the phyllosphere compartments (without the need of them being less abundant in the other compartments), we determined the core genera of each compartment. In this study, we considered a genus to be a core genus of a compartment if it was present in more than 95% of the samples (all time points) of that compartment. To limit the bias due to the presence of contaminants in samples, a genus was considered present in a sample when represented by more than 10 reads, which is a threshold that should filter out 50% of the contaminants based on data from the blanks (Supplementary R code).

### Community dynamics.

To study community dynamics, only the Antwerp samples were used as they represent a regular time series with 10 time points (Table S1). The Bray-Curtis dissimilarities between samples taken in Antwerp of leaves, trunk, and branches were calculated using the data at the ASV level. The time trends of dissimilarities between consecutive time points were tested using the Kendall rank correlation test.

To study the temporal patterns of ASVs on leaves, cooccurring ASVs were clustered using a similar approach as with the Antwerp leaf data set. We repeated the following analysis for 1,000 random rarefactions of the data set to 912 reads/sample (20 of 28 leaf samples remaining). Because of their limited significance, all ASVs with a total abundance of less than 25 reads in this data set were not included in the cooccurrence analysis. At 25 reads, the frequency of ASVs per total read number drastically drops (this threshold is at the elbow in Fig. S3). ASV abundances in samples were scaled by dividing their abundances/sample by their respective total ASV read number in the data set. These scaled data were used for calculating Euclidean distances between ASVs followed by an agglomerative hierarchical clustering procedure using Ward’s method (44). We used a Silhouette plot to determine the optimal number of clusters (45). Based on these results, we divided the hierarchically clustered ASVs into four cooccurrence clusters. The support for each ASV belonging to each of the clusters was expressed as the fraction of times they were found in this cluster over the 1,000 repeated rarefactions. Then, all abundant leaf ASVs—those that made it through the rarefaction and subsequent threshold at least once—were assigned to the cluster with the highest support. The relative abundances of each cluster were visualized through time. Temporal patterns of the clusters were identified, and the clusters were named accordingly: early, middle, late, and general (abundant in all time points).

### ASV overlap between leaves and other compartments.

We estimated which ASVs of the different leaf cooccurrence clusters were shared with other tree phyllosphere compartments. In this presence-absence-based analysis, we tried to minimize the detection of contaminants and again required more than 10 reads for an ASV to be considered present in a sample. We then pooled ASVs per compartment and determined the ASV overlap between the leaves and the other phyllosphere compartments. We separately analyzed overlap for each of the cooccurrence leaf clusters, which were determined by hierarchical clustering as described above. We calculated both the proportion of different leaf ASVs that overlapped with other compartments and the total relative abundances that these ASVs contributed to the leaf communities.
