# Supplementary material for: Bacterial Succession and Community Dynamics of the Emerging Leaf Phyllosphere in Spring
Source: Microbiol Spectr. 2022 Mar 2;10(2):e02420-21. doi: 10.1128/spectrum.02420-21 (PMC8941926; doi:10.1128/spectrum.02420-21)
Supplement: SUPPLEMENTAL FILE 1 — Supplemental material. Download Spectrum02420-21_Supplemental_file1.docx, DOCX file, 4.0 MB [file spectrum02420-21_supplemental_file1.docx]

# Supplementary material

**
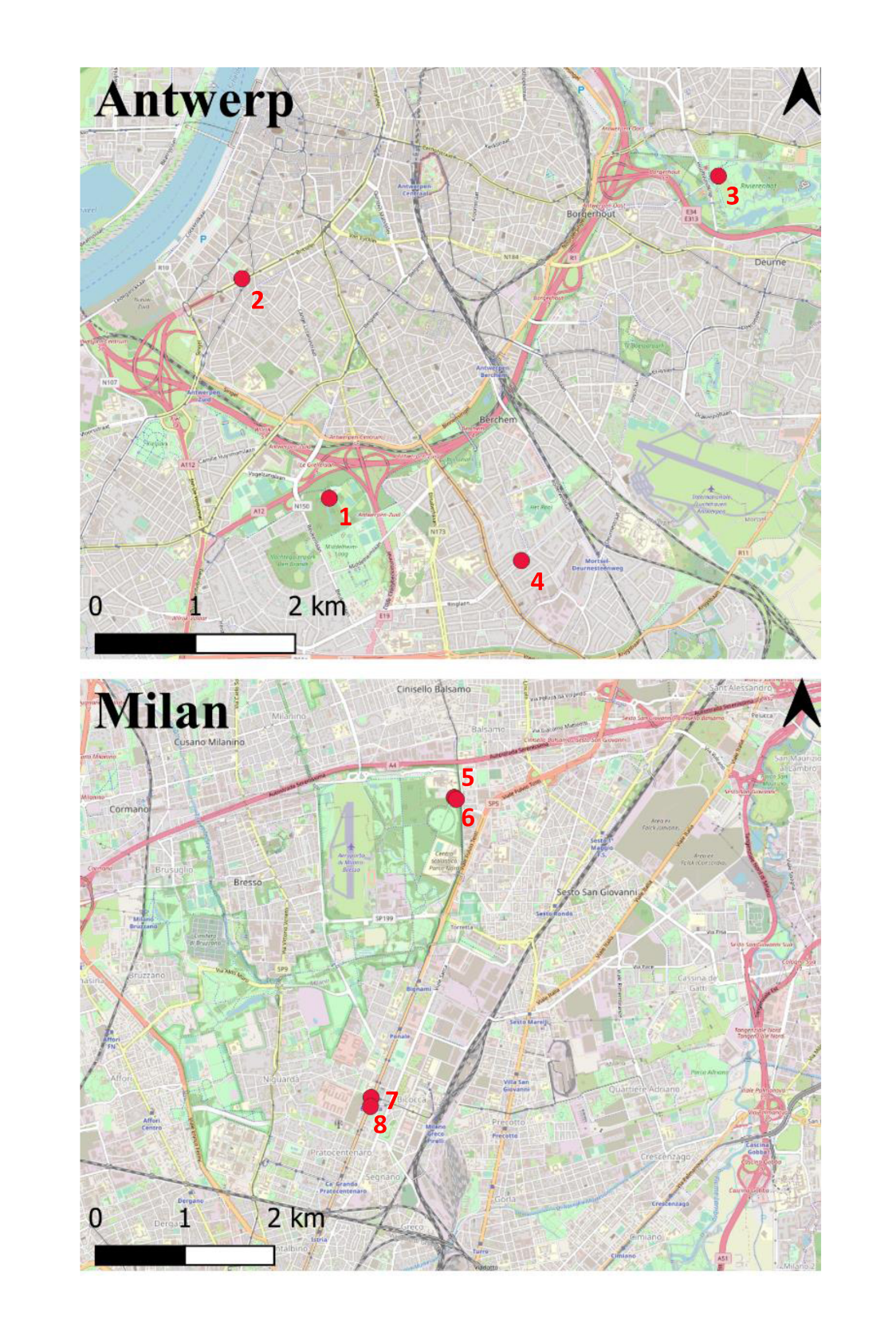
**

# Figure S1: Maps with the locations of the sampled trees indicated with red circles. Four trees were sampled in each city, two right next to busy streets and two in a park. The coordinates of the trees can be found in Table S2.


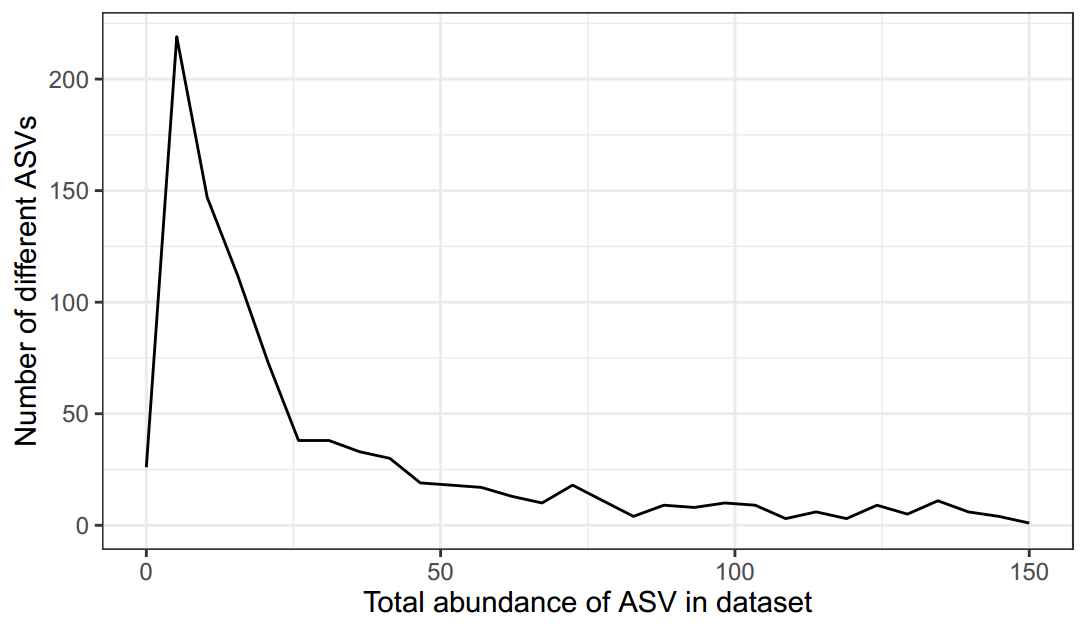


**Figure S2:** This frequency plot represents the total abundances of leaf ASVs of the Antwerp samples. On the x-axis the total amount of reads of an ASV is shown and on the y-axis the amount of ASVs with this abundance is shown. As all ASVs are considered of equal importance in the hierarchical clustering analysis to identify co-occurring ASVs, we discarded the ASVs with lower abundances, in this case those with less than 25 reads. This threshold was set at the elbow point of this graph.


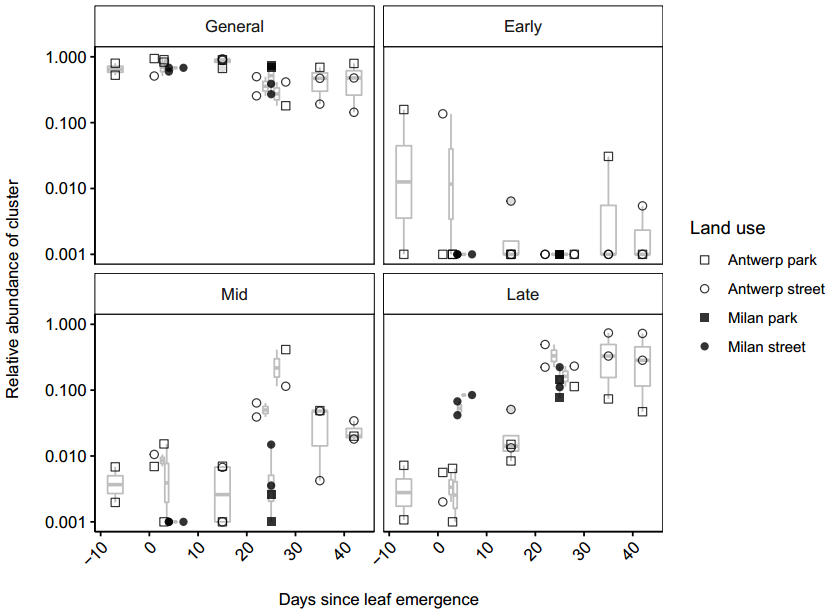


**Figure S3**: Relative abundances (log scale) of different ASV clusters in the leaf phyllosphere over time. The clusters were constructed based on ASV co-occurrences in the Antwerp dataset. Temporal patterns of the clusters were identified and the clusters were named accordingly: early, mid, late, and general (abundant in all time points). Although the Milan samples were not used for identifying the clusters and their members, the relative abundances of the same ASVs in the Milan dataset show remarkably similar temporal trends.


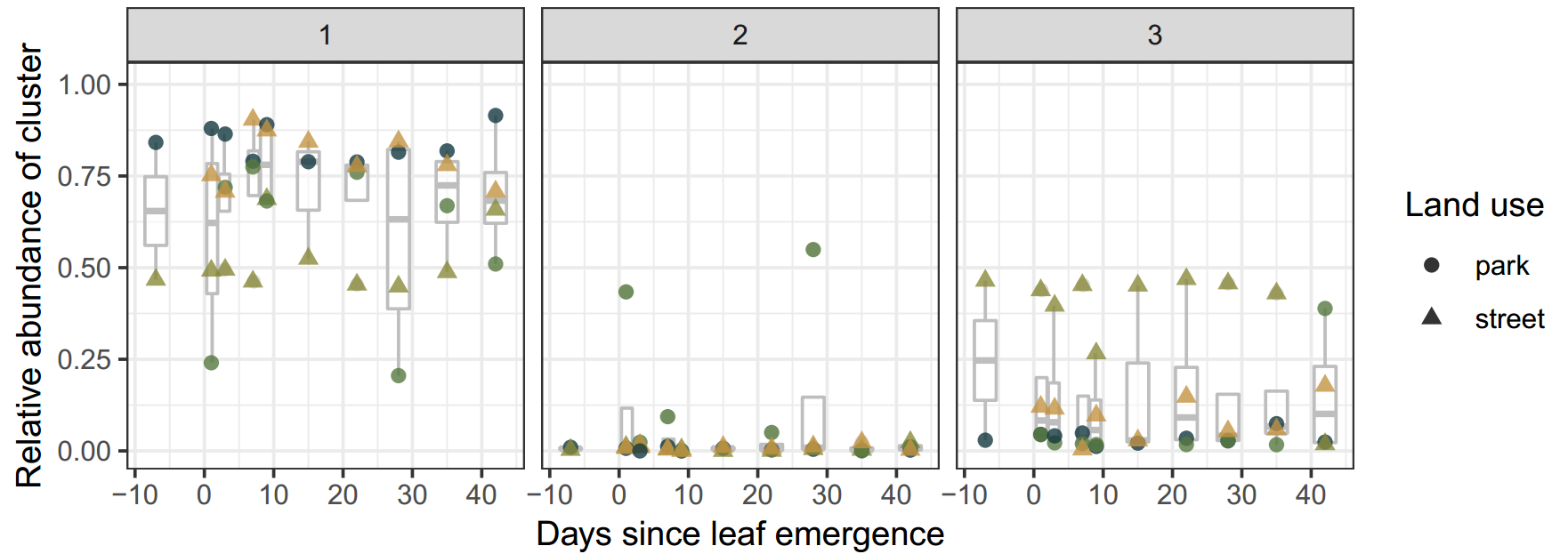


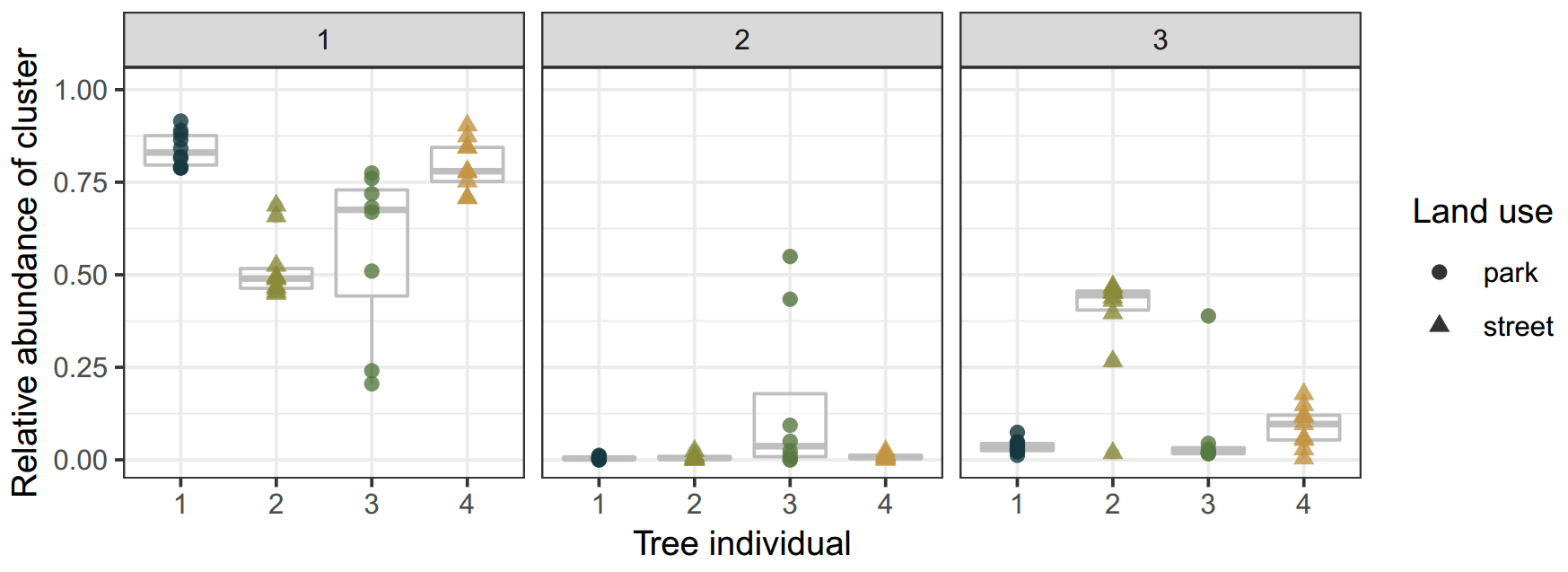


**Figure S4:** ASV co-occurrence analysis was also done for Antwerp trunk samples. According to the repeated (100 rarefactions) silhouette analysis, two or three clusters were most often advised. Each pane shows the total relative abundance of a cluster and every point represents the cluster abundance in a single sample, with the colour of the point referring to the tree individual it was taken. No temporal trend was observed for the co-occurrence clusters of the trunk samples, but the tree from which the trunk was sampled determined the trunk ASV co-occurrences strongly.


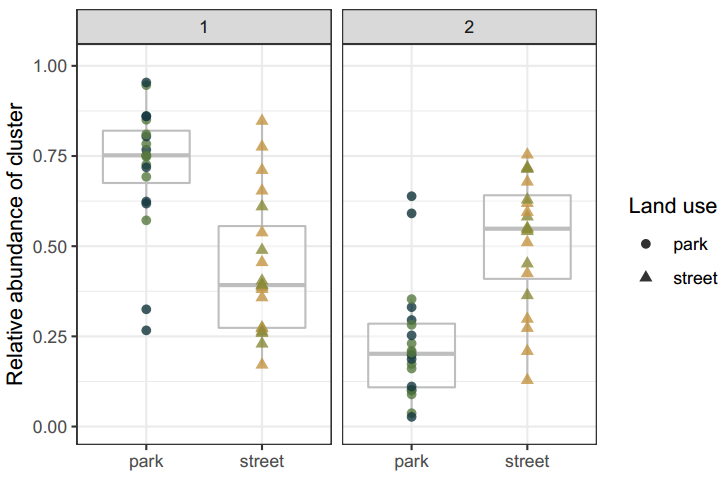

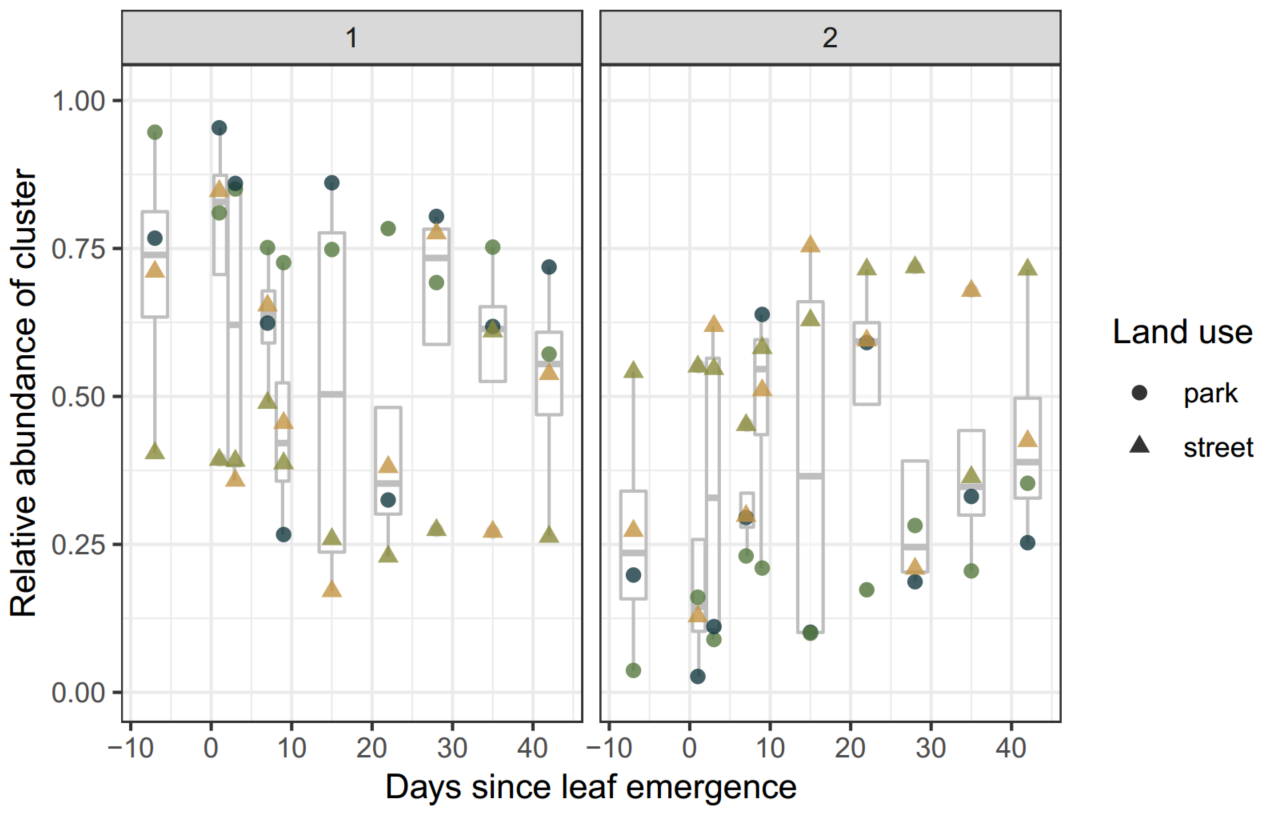


**Figure S5:** ASV co-occurrence analysis was also done for Antwerp branch samples. According to the repeated (100 rarefactions) silhouette analysis, two clusters were most often advised (analysing up to 5 clusters didn't reveal any additional patterns). Each pane shows the total relative abundance of a cluster (number 1 or 2) per sample (park samples green, street samples yellow). Most notable is that the first cluster was most abundant in park samples and the second most abundant in street samples. We observed a temporal pattern: the second cluster slowly but significantly became more abundant over time (Kendall correlation: p=0.044, τ = 0.23). Whenever branch ASVs were also found in the leaves, they usually belonged to the general leaf cluster or to none of the leaf clusters.

**Table S1:** Sampling time points in Antwerp and Milan. Means of weather variables during the sampling period were retrieved from [www.timeanddate.com/weather/](http://www.timeanddate.com/weather/). Weather was rather stable during our study, but city dependent, hence no effect of weather on the bacterial communities could be tested.

| **Sampling date** | **City** | **Weather** | **Temperature (°C)** | **Relative humidity (%)** | **Wind direction** | **Wind speed (km/h)** |
| --- | --- | --- | --- | --- | --- | --- |
| 10/04/2018 | Antwerp | sunny | 12.5 | 80 | SSE | 12 |
| 18/04/2018 | Antwerp | sunny | 14 | 75 | E | 5 |
| 20/04/2018 | Antwerp | sunny | 18.5 | 66 | SW | 7 |
| 24/04/2018 | Antwerp | partly sunny | 10.5 | 78 | SW | 16 |
| 26/04/2018 | Antwerp | partly sunny | 10.5 | 70 | W | 21 |
| 2/05/2019 | Antwerp | sunny | 10 | 62 | S | 16 |
| 9/05/2019 | Antwerp | sunny | 17.5 | 71 | W | 9 |
| 15/05/2019 | Antwerp | sunny | 17.5 | 70 | NNW | 8 |
| 22/05/2019 | Antwerp | sunny | 17.5 | 77 | ENE | 6 |
| 29/05/2018 | Antwerp | sunny | 23.5 | 72 | N | 8 |
| 29/03/2018 | Milan | broken clouds | 11 | 86 | NNE | 8 |
| 3/04/2018 | Milan | broken clouds | 10 | 82 | N | 4 |
| 17/04/2018 | Milan | passing clouds | 19 | 60 | N | 9 |
| 20/04/2018 | Milan | passing clouds | 19 | 60 | NNE | 8 |
| 8/05/2018 | Milan | passing clouds | 21 | 65 | N | 7 |

**Table S2:** Location information of the sampled trees.

| **Tree ID** | **Coordinates** | **City** | **Environment** |
| --- | --- | --- | --- |
| 1 | 51.1873 N 4.4088 E | Antwerp | Park |
| 2 | 51.2070 N 4.3963 E | Antwerp | Street |
| 3 | 51.2162 N 4.4645 E | Antwerp | Park |
| 4 | 51.1817 N 4.4363 E | Antwerp | Street |
| 5 | 45.5452 N 9.2173 E | Milan | Park |
| 6 | 45.5450 N 9.2176 E | Milan | Park |
| 7 | 45.5151 N 9.2054 E | Milan | Street |
| 8 | 45.5142 N 9.2053 E | Milan | Street |

**Table S3:** All ASVs which were found both in the blanks and the samples. Some of them are external contaminants, whereas others likely "leaked" from the samples to the blanks, and are therefore considered cross-contaminants. We decided ASV by ASV to make the distinction. Total relative abundances of ASVs are expressed as their fraction of reads of all reads of the complete dataset.

| **Taxon name** | **Total relative abundance** | **Contamination** |
| --- | --- | --- |
| *Mucilaginibacter 1* | 6.15E-02 | cross |
| *Methylobacterium 1* | 1.27E-02 | cross |
| *Hymenobacter 7* | 8.11E-03 | cross |
| *Spirosoma 1* | 7.74E-03 | cross |
| *Polaromonas 1* | 5.50E-03 | cross |
| *Massilia 4* | 2.44E-03 | cross |
| *Rubellimicrobium 1* | 1.26E-03 | cross |
| *Escherichia 1* | 1.12E-03 | external |
| *Staphylococcus 1* | 7.14E-04 | external |
| *Fimbriimonas 1* | 4.55E-04 | cross |
| *Edaphobacter 4* | 4.04E-04 | cross |
| *Alloiococcus 1* | 3.21E-04 | external |
| *Moraxella 1* | 2.73E-04 | external |
| *Acinetobacter 1* | 2.39E-04 | cross |
| *Streptococcus 1* | 1.54E-04 | external |
| *Fusobacterium 2* | 1.21E-04 | external |
| *Rhizobium 1* | 8.03E-05 | cross |
| *Fusobacterium 1* | 7.56E-05 | external |
| *Moraxella 2* | 7.54E-05 | external |
| *Gemella* | 7.29E-05 | external |
| *Prevotella 1* | 6.28E-05 | external |
| *Microcoleus 2* | 5.60E-05 | external |
| *Chthoniobacteraceae 2* | 2.61E-05 | cross |
| *Alloprevotella* | 2.34E-05 | external |
| *Streptococcus 2* | 1.82E-05 | external |
| *Hymenobacter 49* | 1.64E-05 | cross |
| *Haemophilus 1* | 1.30E-05 | external |
| *Dolosigranulum 1* | 5.15E-06 | external |
| *Citrobacter* | 1.02E-06 | external |

**Table S4:** The ASVs for each of the co-occurrence clusters with their respective support for that cluster and average relative abundance in the Antwerp leaf samples. ASVs are named after their genus (or family if genus was unknown) and their order of abundance within this genus.

|  | **ASV** | **Support** | **Avg abun (%)** | |  | |  | | **ASV** | | **Support** | | **Avg abun (%)** | | |
| --- | --- | --- | --- | --- | --- | --- | --- | --- | --- | --- | --- | --- | --- | --- | --- |
| **General cluster** | |  |  |  | | **Early cluster** | | | |  | |  | | |  |
|  | *Sphingomonas 1* | 1 | 5.7 |  | |  | | *Hymenobacter 29* | | 0.493 | | 0.39 | |  |  |
|  | *EU289441_g 1* | 0.992 | 4.6 |  | |  | | *Sphingomonas 10* | | 0.489 | | 0.2 | |  |  |
|  | *EU289441_g 2* | 0.953 | 4.6 |  | |  | | *Chthoniobacteraceae 3* | | 0.362 | | 0.14 | |  |  |
|  | *Mucilaginibacter 1* | 0.978 | 3.9 |  | |  | | *EU289441_g 8* | | 0.346 | | 0.14 | |  |  |
|  | *EU861940_g 1* | 0.977 | 3.7 |  | |  | | *Sphingomonas 7* | | 0.271 | | 0.13 | |  |  |
|  | *Sphingomonas 2* | 0.998 | 3.5 |  | |  | | *EU289441_g 7* | | 0.208 | | 0.13 | |  |  |
|  | *Beijerinckiaceae 1* | 0.956 | 2.7 |  | |  | | *Hymenobacter 64* | | 0.056 | | 0.1 | |  |  |
|  | *Hymenobacter 1* | 0.972 | 1.4 |  | |  | | *Beijerinckiaceae 3* | | 0.055 | | 0.1 | |  |  |
|  | *Pseudomonas 1* | 0.974 | 1.1 |  | |  | | *Edaphobacter 2* | | 0.046 | | 0.1 | |  |  |
|  | *Hymenobacter 15* | 0.972 | 1 |  | |  | | *Nocardioides 1* | | 0.001 | | 0.09 | |  |  |
|  | *Methylobacterium 1* | 0.716 | 1 |  | |  | | *Planctomycetaceae 2* | | 0.002 | | 0.08 | |  |  |
|  | *Pseudomonas 3* | 0.884 | 0.9 |  | |  | | *Edaphobacter 13* | | 0.003 | | 0.07 | |  |  |
|  | *EU289441_g 3* | 0.912 | 0.8 |  | |  | | *Hymenobacter 59* | | 0.001 | | 0.07 | |  |  |
|  | *Methylobacterium 3* | 0.765 | 0.8 |  | | **Mid cluster** | | | |  | |  | |  |  |
|  | *Polaromonas 1* | 0.883 | 0.8 |  | |  | | *Chamaesiphon 1* | | 0.913 | | 0.64 | |  |  |
|  | *EU861940_g 2* | 0.977 | 0.7 |  | |  | | *Sphingomonas 16* | | 0.913 | | 0.51 | |  |  |
|  | *Rhizobacter 1* | 0.632 | 0.7 |  | |  | | *Paracoccus 2* | | 0.817 | | 0.42 | |  |  |
|  | *Massilia 1* | 0.978 | 0.7 |  | |  | | *Cronobacter 1* | | 0.913 | | 0.32 | |  |  |
|  | *Bacteria 1* | 0.953 | 0.6 |  | |  | | *Thermomonas* | | 0.894 | | 0.31 | |  |  |
|  | *Comamonadaceae 1* | 0.977 | 0.6 |  | |  | | *Intrasporangiaceae 1* | | 0.913 | | 0.28 | |  |  |
|  | *EU289441_g 4* | 0.912 | 0.6 |  | |  | | *Qipengyuania* | | 0.913 | | 0.27 | |  |  |
|  | *Methylobacterium 2* | 0.97 | 0.6 |  | |  | | *Pseudarthrobacter* | | 0.782 | | 0.24 | |  |  |
|  | *Hymenobacter 9* | 0.971 | 0.6 |  | |  | | *Bacillus 3* | | 0.533 | | 0.23 | |  |  |
|  | *EU861940_g 3* | 0.774 | 0.5 |  | |  | | *Acetobacteraceae 3* | | 0.911 | | 0.22 | |  |  |
|  | *Hymenobacter 6* | 0.962 | 0.5 |  | |  | | *Pleurocapsa 1* | | 0.568 | | 0.14 | |  |  |
|  | *EU861940_g 4* | 0.75 | 0.4 |  | |  | | *Sphingomonadaceae 4* | | 0.398 | | 0.13 | |  |  |
|  | *Sphingomonas 13* | 0.813 | 0.4 |  | |  | | *Rhodobacteraceae 1* | | 0.322 | | 0.13 | |  |  |
|  | *Hymenobacter 4* | 0.841 | 0.4 |  | |  | | *Enterobacter 2* | | 0.052 | | 0.11 | |  |  |
|  | *Pseudomonas 2* | 0.884 | 0.4 |  | |  | | *Oscillatoria_g3 1* | | 0.001 | | 0.09 | |  |  |
|  | *Sphingomonas 3* | 0.905 | 0.4 |  | |  | | *Sporosarcina 1* | | 0.001 | | 0.08 | |  |  |
|  | *Methylobacterium 4* | 0.97 | 0.4 |  | |  | | *AB374370_g 1* | | 0.003 | | 0.07 | |  |  |
|  | *AM697144_g 1* | 0.977 | 0.4 |  | | **Late cluster** | | | |  | |  | |  |  |
|  | *AY796037_g 1* | 0.978 | 0.4 |  | |  | | *Massilia 2* | | 0.604 | | 2.1 | |  |  |
|  | *Spirosoma 1* | 0.753 | 0.4 |  | |  | | *Bacillus 1* | | 0.786 | | 1.07 | |  |  |
|  | *Hymenobacter 2* | 0.765 | 0.4 |  | |  | | *Erwinia 2* | | 0.414 | | 1.05 | |  |  |
|  | *EU861940_g 8* | 0.92 | 0.4 |  | |  | | *Skermanella 1* | | 0.787 | | 0.86 | |  |  |
|  | *HQ674891_g 2* | 0.91 | 0.4 |  | |  | | *Pantoea* | | 0.778 | | 0.85 | |  |  |
|  | *Sphingomonadaceae 2* | 0.972 | 0.35 |  | |  | | *Rubellimicrobium 1* | | 0.784 | | 0.8 | |  |  |
|  | *Massilia 6* | 0.884 | 0.33 |  | |  | | *Clostridium 1* | | 0.603 | | 0.77 | |  |  |
|  | *Hymenobacter 17* | 0.908 | 0.33 |  | |  | | *Noviherbaspirillum* | | 0.646 | | 0.74 | |  |  |
|  | *Sphingosinicella 1* | 0.768 | 0.31 |  | |  | | *ALVU_g 1* | | 0.787 | | 0.63 | |  |  |
|  | *Hymenobacter 5* | 0.977 | 0.31 |  | |  | | *Blastococcus 1* | | 0.778 | | 0.54 | |  |  |
|  | *Sphingomonadaceae 3* | 0.768 | 0.3 |  | |  | | *Lactobacillus 1* | | 0.778 | | 0.54 | |  |  |
|  | *Sphingomonas 5* | 0.967 | 0.28 |  | |  | | *Paracoccus 1* | | 0.787 | | 0.54 | |  |  |
|  | *Polaromonas 2* | 0.974 | 0.27 |  | |  | | *Arthrobacter* | | 0.753 | | 0.47 | |  |  |
|  | *Sediminibacterium 1* | 0.771 | 0.27 |  | |  | | *Marmoricola 1* | | 0.787 | | 0.46 | |  |  |
|  | *Sphingobacteriaceae 1* | 0.877 | 0.26 |  | |  | | *Massilia 4* | | 0.778 | | 0.45 | |  |  |
|  | *Edaphobacter 3* | 0.77 | 0.26 |  | |  | | *ALVU_g 2* | | 0.735 | | 0.43 | |  |  |
|  | *Edaphobacter 6* | 0.977 | 0.26 |  | |  | | *Brevundimonas 1* | | 0.556 | | 0.41 | |  |  |
|  | *DQ532271_g 2* | 0.488 | 0.25 |  | |  | | *Erythrobacteraceae* | | 0.789 | | 0.41 | |  |  |
|  | *Hymenobacter 16* | 0.972 | 0.24 |  | |  | | *Sphingomonas 33* | | 0.788 | | 0.32 | |  |  |
|  | *Deinococcus 1* | 0.938 | 0.24 |  | |  | | *Truepera 1* | | 0.787 | | 0.31 | |  |  |
|  | *Spirosoma 3* | 0.972 | 0.23 |  | |  | | *Modestobacter 1* | | 0.778 | | 0.29 | |  |  |
|  | *Hymenobacter 11* | 0.964 | 0.23 |  | |  | | *Enterobacter 1* | | 0.788 | | 0.28 | |  |  |
|  | *Edaphobacter 5* | 0.833 | 0.23 |  | |  | | *Deinococcus 2* | | 0.781 | | 0.27 | |  |  |
|  | *DQ490355_g 1* | 0.767 | 0.22 |  | |  | | *HQ910322_g 2* | | 0.785 | | 0.25 | |  |  |
|  | *EU861847_g 2* | 0.763 | 0.21 |  | |  | | *Deinococcus 4* | | 0.786 | | 0.23 | |  |  |
|  | *JPOM_g 2* | 0.662 | 0.21 |  | |  | | *Chroococcidiopsis_f 1* | | 0.767 | | 0.18 | |  |  |
|  | *Sphingomonas 12* | 0.913 | 0.21 |  | |  | | *Oxalobacteraceae 1* | | 0.778 | | 0.18 | |  |  |
|  | *Nakamurella 1* | 0.87 | 0.21 |  | |  | | *Bradyrhizobium 1* | | 0.558 | | 0.17 | |  |  |
|  | *Hymenobacter 8* | 0.865 | 0.2 |  | |  | | *Sphingomonas 50* | | 0.716 | | 0.16 | |  |  |
|  | *Clostridium 2* | 0.809 | 0.2 |  | |  | | *Terrisporobacter 1* | | 0.276 | | 0.16 | |  |  |
|  | *Sphingomonas 26* | 0.905 | 0.2 |  | |  | | *Aquabacterium 1* | | 0.596 | | 0.15 | |  |  |
|  | *Kurthia 1* | 0.605 | 0.19 |  | |  | | *Roseomonas 1* | | 0.578 | | 0.14 | |  |  |
|  | *Sphingomonadaceae 1* | 0.678 | 0.19 |  | |  | | *Roseomonas 2* | | 0.474 | | 0.14 | |  |  |
|  | *Lactobacillus 2* | 0.901 | 0.18 |  | |  | | *DQ914863_g* | | 0.287 | | 0.13 | |  |  |
|  | *Chlamydiales 1* | 0.868 | 0.18 |  | |  | | *Hafnia* | | 0.075 | | 0.11 | |  |  |
|  | *HQ674891_g 1* | 0.455 | 0.18 |  | |  | | *EU753646_g 1* | | 0.046 | | 0.11 | |  |  |
|  | *Erwinia 1* | 0.87 | 0.17 |  | |  | |  | |  | |  | |  |  |
|  | *JPOM_g 1* | 0.587 | 0.17 |  | |  | |  | |  | |  | |  |  |
|  | *AY796037_g 2* | 0.61 | 0.16 |  | |  | |  | |  | |  | |  |  |
|  | *Pseudomonas 5* | 0.724 | 0.16 |  | |  | |  | |  | |  | |  |  |
|  | *Turicibacter 1* | 0.835 | 0.16 |  | |  | |  | |  | |  | |  |  |
|  | *Romboutsia 1* | 0.729 | 0.16 |  | |  | |  | |  | |  | |  |  |
|  | *Variovorax 1* | 0.67 | 0.15 |  | |  | |  | |  | |  | |  |  |
|  | *Sphingorhabdus 2* | 0.502 | 0.14 |  | |  | |  | |  | |  | |  |  |
|  | *Edaphobacter 1* | 0.271 | 0.14 |  | |  | |  | |  | |  | |  |  |
|  | *HQ674891_g 8* | 0.411 | 0.13 |  | |  | |  | |  | |  | |  |  |
|  | *Enterobacteriaceae 4* | 0.339 | 0.13 |  | |  | |  | |  | |  | |  |  |
|  | *EU861940_g 6* | 0.25 | 0.13 |  | |  | |  | |  | |  | |  |  |
|  | *Edaphobacter 15* | 0.289 | 0.13 |  | |  | |  | |  | |  | |  |  |
|  | *AB128886_g* | 0.134 | 0.12 |  | |  | |  | |  | |  | |  |  |
|  | *Hymenobacter 7* | 0.175 | 0.12 |  | |  | |  | |  | |  | |  |  |
|  | *JPOM_g 3* | 0.129 | 0.11 |  | |  | |  | |  | |  | |  |  |
|  | *Sphingomonas 23* | 0.11 | 0.11 |  | |  | |  | |  | |  | |  |  |
|  | *Hymenobacter 40* | 0.106 | 0.11 |  | |  | |  | |  | |  | |  |  |
|  | *Hymenobacter 114* | 0.027 | 0.11 |  | |  | |  | |  | |  | |  |  |
|  | *Aureimonas 1* | 0.021 | 0.11 |  | |  | |  | |  | |  | |  |  |
|  | *DQ532271_g 1* | 0.054 | 0.1 |  | |  | |  | |  | |  | |  |  |
|  | *Flavobacterium 2* | 0.013 | 0.1 |  | |  | |  | |  | |  | |  |  |
|  | *Alkanindiges 1* | 0.029 | 0.1 |  | |  | |  | |  | |  | |  |  |
|  | *Hymenobacter 20* | 0.062 | 0.1 |  | |  | |  | |  | |  | |  |  |
|  | *Nocardioides 3* | 0.013 | 0.1 |  | |  | |  | |  | |  | |  |  |
|  | *Kineococcus 1* | 0.024 | 0.1 |  | |  | |  | |  | |  | |  |  |
|  | *HQ674891_g 6* | 0.025 | 0.1 |  | |  | |  | |  | |  | |  |  |
|  | *Chthoniobacteraceae 1* | 0.022 | 0.1 |  | |  | |  | |  | |  | |  |  |
|  | *Comamonadaceae 6* | 0.003 | 0.09 |  | |  | |  | |  | |  | |  |  |
|  | *Sphingomonas 6* | 0.008 | 0.09 |  | |  | |  | |  | |  | |  |  |
|  | *Nakamurella 2* | 0.013 | 0.09 |  | |  | |  | |  | |  | |  |  |
|  | *HE587193_g 1* | 0.011 | 0.09 |  | |  | |  | |  | |  | |  |  |
|  | *Chthoniobacteraceae 4* | 0.01 | 0.09 |  | |  | |  | |  | |  | |  |  |
|  | *Barrientosiimonas* | 0.013 | 0.09 |  | |  | |  | |  | |  | |  |  |
|  | *Sphingomonas 55* | 0.008 | 0.09 |  | |  | |  | |  | |  | |  |  |
|  | *Ensifer* | 0.001 | 0.09 |  | |  | |  | |  | |  | |  |  |
|  | *Edaphobacter 16* | 0.007 | 0.09 |  | |  | |  | |  | |  | |  |  |
|  | *AY796037_g 3* | 0.008 | 0.09 |  | |  | |  | |  | |  | |  |  |
|  | *Hymenobacter 66* | 0.002 | 0.08 |  | |  | |  | |  | |  | |  |  |
|  | *Hymenobacter 14* | 0.004 | 0.08 |  | |  | |  | |  | |  | |  |  |
|  | *Edaphobacter 4* | 0.003 | 0.08 |  | |  | |  | |  | |  | |  |  |
|  | *Hymenobacter 34* | 0.003 | 0.07 |  | |  | |  | |  | |  | |  |  |
|  | *Hymenobacter 37* | 0.001 | 0.07 |  | |  | |  | |  | |  | |  |  |
|  | *EU289441_g 6* | 0.001 | 0.07 |  | |  | |  | |  | |  | |  |  |
|  | *Hymenobacter 67* | 0.001 | 0.07 |  | |  | |  | |  | |  | |  |  |
|  | *EU861940_g 9* | 0.001 | 0.06 |  | |  | |  | |  | |  | |  |  |
|  | *Sphingomonas 8* | 0.001 | 0.06 |  | |  | |  | |  | |  | |  |  |

**Supplementary R code**

To make this code work for you:

- Also download the .Robject from figshare: https://figshare.com/s/d42cb6524fa512e408d9

- Copy all content of this file to an R script and save with file extension .Rmd

---

title: "Bacterial succession in the phyllosphere"

author: "Wenke"

date: "13-12-2021"

output: html_document

---

```{r setup, include=FALSE}

knitr::opts_chunk$set(echo = TRUE, message = F, warning = F, fig.width = 10)

```

### Setting up the workspace

```{r}

#devtools::install_github("SWittouck/tidyamplicons") # need R 3.6

library(tidyverse)

library(glue)

library(tidyamplicons)

# Adapted tidyamplicons function to use taxon name instead of taxon ID:

as_abundances_matrix <- function(abundances, value = abundance) {

if (

! is.data.frame(abundances) |

is.null(abundances$taxon_name) |

is.null(abundances$sample_id)

) stop("need abundances in right format")

value <- enquo(value)

abundances_wide <- abundances %>%

select(sample_id, taxon_name, !! value) %>%

spread(key = taxon_name, value = !! value, fill = 0)

abundances_wide %>%

select(- sample_id) %>%

as.matrix() %>%

`row.names<-`(abundances_wide$sample_id)

}

area_plot <- function(ta, n = 12, x = sample_clustered, geom_area = T) {

# convert promise to formula

x <- substitute(x)

# add sample_clustered if not present

if (! "sample_clustered" %in% names(ta$samples)) {

ta <- add_sample_clustered(ta)

}

# add taxon_name_color if not present

if (! "taxon_name_color" %in% names(ta$taxa)) {

ta <- add_taxon_name_color(ta, n = n)

}

ta <- ta %>%

select_taxa(taxon_id,taxon_name_color)%>%

aggregate_taxa()

# add relative abundances if not present

if (! "rel_abundance" %in% names(ta$abundances)) {

ta <- add_rel_abundance(ta)

}

saminfo<-ta$samples%>%

select(sample_id,tree_id,type,days_since_leaf_emergence)

all<-expand.grid(sample_id = unique(ta$samples$sample_id),

taxon_name_color = unique(ta$taxa$taxon_name_color))%>%

left_join(saminfo)

ta<-left_join(all,get_abundances_extended(ta))%>%

replace_na(list(rel_abundance=0))

# make plot and return

plot <- ta %>%

ggplot(aes_(x = x, y = ~rel_abundance, fill = ~taxon_name_color)) +

scale_fill_brewer(palette = "Paired", name = "Taxon") +

xlab("sample") + ylab("relative abundance") +

theme(

#axis.text.x = element_text(angle = 90),

#axis.ticks.x = element_blank(),

panel.background = element_rect(fill = 'white', colour = 'white')

)

# add geom_bar if requested

if (geom_area) {

plot <- plot + geom_area()

}

plot

}

# Set your working directory

setwd("...")

```

### Load data

```{r,fig.height = 5}

load("run.Robject")

run<-run%>%

add_taxon_name()

```

### Overview data

```{r}

"ASV"

numbers(run)

"Trunk"

runt<-filter_samples(run, type=="Trunk")

n<-numbers(runt)

n[3]/n[1]

"Buds"

runbd<-filter_samples(run, type=="Buds")

n<-numbers(runbd)

n[3]/n[1]

"Leaves"

runl<-filter_samples(run, type=="Leaves")

n<-numbers(runl)

n[3]/n[1]

"Branch"

runb<-filter_samples(run, type=="Branch")

n<-numbers(runb)

n[3]/n[1]

"genus"

rg<-aggregate_taxa(run,rank="genus")

numbers(rg)

"family"

rf<-aggregate_taxa(run,rank="family")

numbers(rf)

"phylum"

rp<-aggregate_taxa(run,rank="phylum")

numbers(rp)

#View(rp$taxa)

rm(rp,rf,rg,n,runbd,runl,runb,runt)

```

### Checking blanks and removing contaminants

```{r}

runall<-run%>%

add_lib_size()

runb<-runall%>%

filter_samples(type=="KIT")

nrow(runb$abundances)/nrow(runb$samples) #avg ASVs per blank sample

runs<-runall%>%

filter_samples(type!="KIT")%>%

add_max_rel_abundance()%>%

add_total_rel_abundance()

# taxa both in controls and samples:

cc<-intersect(runb$taxa$taxon_name,runs$taxa$taxon_name)

# samples with only common taxa:

runc<-filter_taxa(runs,taxon_name %in% cc)

# read numbers of these taxa:

tn<-select(runc$taxa,taxon_name,taxon_id,max_rel_abundance,total_rel_abundance)

new<-left_join(runc$abundances,tn)

# --> potential contaminants in samples (judged so if they were present in blanks and not very abundant in samples, or abundant in other samples of same run, hence not phyllosphere-specific):

# Alloiococcus 1 (max 31 reads)

# Alloprevotella (once 10 reads)

# Citrobacter (max 9 reads)

# Dolosigranulum 1 (max 7 reads)

# Fusobacterium 1 (max 15 reads)

# Fusobacterium 2 (max 18 reads)

# Gemella (max 8 reads)

# Haemophilus 1 (max 14 reads)

# Microcoleus 2 (once, 12 reads)

# Moraxella 1 (max 38 reads)

# Moraxella 2 (max 9 reads)

# Prevotella 1 (max 31 reads)

# Streptococcus 1 (max 27 reads)

# Streptococcus 2 (once 14 reads)

#Escherichia 1 (max 112 reads)

#Staphylococcus 1 (max 61 reads)

# in blank but probably no contaminant: Acinetobacter 1, Mucilaginibacter 1, Methylobacterium 1, Massilia 4 (max 492), Edaphobacter 4 (max 320 reads), Hymenobacter 7, Fimbriimonas 1, Polaromonas 1, Spirosoma 1 (in data also Hymenobacter 49,Chthoniobacteraceae 2, Rubellimicrobium 1, Rhizobium 1 of doubting cases)

# doubting case: -Chthoniobacteraceae 2 (max 140 reads), Escherichia 1 (max 112 reads), -Hymenobacter 49 (max 88 reads), -Rhizobium 1 (max 52), -Rubellimicrobium 1 (max 131), Staphylococcus 1 (max 61 reads, in many samples)

#Specific ASVs were identified as contaminants based on their presence in the blanks in combination with their low presence in the phyllosphere samples (less than 70 reads), and their likelihood to be common contaminants (Supplementary table 1).

nc<-c("Acinetobacter 1","Mucilaginibacter 1", "Methylobacterium 1", "Massilia 4", "Edaphobacter 4", "Hymenobacter 7", "Fimbriimonas 1", "Polaromonas 1", "Spirosoma 1","Hymenobacter 49","Chthoniobacteraceae 2","Rubellimicrobium 1","Rhizobium 1") #non-external-contaminants

# reads of external contaminants in samples

ec<-setdiff(cc,nc)

new<-filter(new,new$taxon_name %in% ec)

ggplot(new,aes(x=abundance))+

geom_histogram(bins=50)+

xlim(0,100)

quantile(new$abundance,probs = c(0, 0.1, 0.25, 0.5,0.75,0.9,0.95,0.99))

cleantaxa<-setdiff(runall$taxa$taxon_name, ec)

runclean<-run%>%

filter_taxa(taxon_name %in% cleantaxa)

numbers(run)[3]

1-numbers(runclean)[3]/numbers(run)[3]

length(ec)

run<-runclean%>%

filter_samples(type!="KIT")

rm(runall,runc,runs,runb,tn,ec,nc,cc,new,cleantaxa,runclean)

```

### Checking cities and their overlap

```{r}

all<-run#%>%

#aggregate_taxa(rank="genus")

"Antwerp"

antw<-all%>%

filter_samples(run=="Antwerp")

numbers(antw)

"Milan"

mil<-all%>%

filter_samples(run=="Milan")

numbers(mil)

"Both cities"

numbers(all)

"ASV/Genus level intersection Antwerp-Milan"

i<-intersect(antw$taxa$taxon_name,mil$taxa$taxon_name)

length(i)

length(i)/nrow(all$taxa)

rm(all,mil,antw,i)

```

### Most abundant taxa and Figure 1A

```{r}

library(vegan)

rung<-run%>%

aggregate_taxa(rank="genus")%>%

mutate_samples(tree_id=paste("Tree",tree_id,"_",environment,sep="")) %>%

add_total_rel_abundance()%>%

add_taxon_name_color()

rung$samples$tree_id<-as.character(rung$samples$tree_id)

View(rung$taxa)

# Exploratory figures

runglima<-rung%>%

filter_samples(run=="Antwerp")%>%

add_rel_abundance()%>%

mutate_abundances(abundance=rel_abundance)%>% # making sure all samples are weighed equally within one group

select_samples(sample_id,run,type,tree_id)%>%

aggregate_samples() # grouping samples

runglimm<-rung%>%

filter_samples(run=="Milan")%>%

add_rel_abundance()%>%

mutate_abundances(abundance=rel_abundance)%>% # making sure all samples are weighed equally within one group

select_samples(sample_id,run,type,tree_id)%>%

aggregate_samples() # grouping samples

bar_plot(runglima)+

geom_point(size=2.5,aes(y = - 0.025, col = type,shape=tree_id))+

scale_colour_manual(values=c("darkorange3","darkgreen","chartreuse3","darkorange4"))+

scale_shape_manual(values=c(15,16,17,18),guide = FALSE)+

xlab("")+

labs(col = "Tree compartment")+

theme(axis.title.x=element_blank(),axis.text.x=element_blank())

bar_plot(runglimm)+

geom_point(size=2.5,aes(y = - 0.025, col = type,shape=tree_id))+

scale_colour_manual(values=c("darkorange3","darkgreen","chartreuse3","darkorange4"))+

scale_shape_manual(values=c(15,16,17,18),guide = FALSE)+

xlab("")+

labs(col = "Tree compartment")+

theme(axis.title.x=element_blank(),axis.text.x=element_blank())

# Figure 1

runglima<-rung%>%

filter_samples(run=="Antwerp")%>%

mutate_samples(type=ifelse(type=="Buds", "Leaves",type)) %>%

add_rel_abundance()%>%

mutate_abundances(abundance=rel_abundance)%>% # making sure all samples are weighed equally within one group

select_samples(sample_id,run,type,tree_id,days_since_leaf_emergence)%>%

aggregate_samples() # grouping samples

#runglima$samples$days_since_leaf_emergence<-as.factor(runglima$samples$days_since_leaf_emergence)

runglimm<-rung%>%

filter_samples(run=="Milan")%>%

mutate_samples(type=ifelse(type=="Buds", "Leaves",type)) %>%

add_rel_abundance()%>%

mutate_abundances(abundance=rel_abundance)%>% # making sure all samples are weighed equally within one group

select_samples(sample_id,run,type,tree_id,days_since_leaf_emergence)%>%

aggregate_samples() # grouping samples

#runglimm$samples$days_since_leaf_emergence<-as.factor(runglimm$samples$days_since_leaf_emergence)

area_plot(runglima,x=days_since_leaf_emergence)+

facet_grid(tree_id~type)+

xlab("Days since leaf emergence")+

theme(axis.text.x = element_text(size=7))+

theme(axis.text.y = element_text(size=7))

area_plot(runglimm,x=days_since_leaf_emergence)+

facet_grid(tree_id~type)+

xlab("Days since leaf emergence")+

theme(axis.text.x = element_text(size=7))+

theme(axis.text.y = element_text(size=7))

rm(runglima,runglimm,rung)

detach("package:vegan", unload=TRUE)

```

### Indicator taxa using clr transformed data

```{r}

library(permute)

library(indicspecies)

library(compositions)

set.seed(1234)

runi<-run%>%

aggregate_taxa(rank='genus')%>%

add_taxon_name()%>%

filter_samples(days_since_leaf_emergence%in%c(42))%>%

select_taxa(taxon_id,taxon_name)%>%

select_samples(sample_id,type)

runi$abundances<-left_join(runi$abundances,runi$taxa)

asv<-as.data.frame(as_abundances_matrix(runi$abundances))

#asv[1:5,1:5]

m<-matrix(nrow=0,ncol=ncol(asv))

for (i in seq(1,nrow(asv),1)){

transformed<-clr(asv[i,])

m<-rbind(m,transformed)

}

rownames(m)<-rownames(asv)

#m[1:5,1:5]

m<-as.data.frame(m)

(groups<-runi$samples[order(runi$samples$sample_id, decreasing = FALSE),])

FALSE %in% (rownames(m)==groups$sample_id)# should be false

indval = multipatt(m, groups$type, control = how(nperm=999))

summary(indval, indvalcomp=TRUE)

rm(indval,groups,i, m, asv,runi,transformed)

```

### Core community

obviously, core taxa differ slightly when considering cities separately, environments seperately and their interactions seperately. Deleted code for space considerations.

```{r}

runf<-run%>%

aggregate_taxa(rank="genus")%>%

add_taxon_name()%>%

add_rel_abundance()%>%

filter_abundances(abundance>10)

taxocc<-matrix(nrow=0,ncol=8)

comp<-c("Buds","Leaves","Trunk","Branch")

for (i in seq(1,4,1)){

runb<-runf%>%

filter_samples(type==comp[i])%>%

add_rel_occurrence()%>%

mutate_taxa(occ=rel_occurrence)%>%

add_mean_rel_abundances()%>%

mutate_taxa(mean_rel_abundances=round(mean_rel_abundance,2))%>%

select_taxa(class,family,genus,taxon_id,occ,mean_rel_abundances)

runb2<-runb$taxa%>%

filter(occ>0.9499)

compartment<-cbind(rep(comp[i],length(runb2$occ)),rep("Both",length(runb2$occ)))

runb2<-cbind(runb2,compartment)

taxocc<-rbind(taxocc,runb2)

}

city<-c("Antwerp","Milan")

for (j in seq(1,2,1)){

for (i in seq(1,4,1)){

runb<-runf%>%

filter_samples(type==comp[i])%>%

filter_samples(run==city[j])%>%

add_rel_occurrence()%>%

mutate_taxa(occ=rel_occurrence)%>%

add_mean_rel_abundances()%>%

mutate_taxa(mean_rel_abundances=round(mean_rel_abundance,2))%>%

select_taxa(class,family,genus,taxon_id,occ,mean_rel_abundances)

runb2<-runb$taxa%>%

filter(occ>0.9499)

compartment<-cbind(rep(comp[i],length(runb2$occ)),rep(city[j],length(runb2$occ)))

runb2<-cbind(runb2,compartment)

taxocc<-rbind(taxocc,runb2)

}

}

tax<-unique(taxocc$taxon_id)

taxall<-matrix(nrow=0,ncol=7)

for (i in seq(1,4,1)){

runb<-runf%>%

filter_samples(type==comp[i])%>%

add_rel_occurrence()%>%

mutate_taxa(occ=rel_occurrence)%>%

add_mean_rel_abundances()%>%

mutate_taxa(mean_rel_abundances=round(mean_rel_abundance,2))%>%

select_taxa(class,family,genus,taxon_id,occ,mean_rel_abundances)

runb2<-runb$taxa%>%

filter(taxon_id%in%tax)

compartment<-rep(paste(comp[i],"Both",sep="_"),length(runb2$taxon_id))

runb2<-cbind(runb2,compartment)

taxall<-rbind(taxall,runb2)

}

for (j in seq(1,2,1)){

for (i in seq(1,4,1)){

runb<-runf%>%

filter_samples(type==comp[i])%>%

filter_samples(run==city[j])%>%

add_rel_occurrence()%>%

mutate_taxa(occ=rel_occurrence)%>%

add_mean_rel_abundances()%>%

mutate_taxa(mean_rel_abundances=round(mean_rel_abundance,2))%>%

select_taxa(class,family,genus,taxon_id,occ,mean_rel_abundances)

runb2<-runb$taxa%>%

filter(taxon_id%in%tax)

compartment<-rep(paste(comp[i],city[j],sep="_"),length(runb2$taxon_id))

runb2<-cbind(runb2,compartment)

taxall<-rbind(taxall,runb2)

}

}

taxabun<-runf%>%

add_total_rel_abundance()

taxabun<-taxabun$taxa%>%

select(taxon_id,total_rel_abundance)

core<-taxall%>%

select(-occ)%>%

spread(compartment,mean_rel_abundances)%>%

left_join(taxabun)%>%

arrange(desc(total_rel_abundance))

core2<-taxall%>%

select(-mean_rel_abundances)%>%

spread(compartment,occ)%>%

left_join(taxabun)%>%

arrange(desc(total_rel_abundance))

write.table(core,"coretaxa.txt",sep="\t")

write.table(core2,"coretaxa2.txt",sep="\t")

rm(core,core2,taxocc,runb,runb2,runf,taxabun,taxall,tax,city,comp,compartment,i,j)

```

### Both cities PCOA (genus): Figure 1B

Also checked with Jaccard: not very different

```{r,fig.height = 5}

rungen<-run%>%

aggregate_taxa(rank='genus')%>%

mutate_samples(Location=str_c(run,environment,sep=" "))%>%

add_pcoa()

ggplot(rungen$samples, aes(x = pcoa1, y = pcoa2, col = type))+

geom_point(size=2,aes(shape=Location)) +

#geom_text(label=rungen$samples$lib_size,size=2,vjust=1.2,hjust=-0.6)+

scale_colour_manual(values=c("darkorange3","darkgreen","chartreuse3","darkorange4"))+

scale_shape_manual(values=c(8,16,7,15))+

stat_ellipse(size=0.6,alpha=0.7)+

labs(col = "Compartment") +

theme_bw()+

theme(panel.grid.major = element_blank(), panel.grid.minor = element_blank())

rm(rungen)

```

### Permanova

for both cities (best at genus level): Table 1

no big differences with Hellinger transformation

```{r}

rung<-run%>%

aggregate_taxa(rank="genus")

matrix<-get_rel_abundance_matrix(rung)

dis<- vegan::vegdist(matrix, method = "bray")

groups1<-rung$samples$type

groups2<-rung$samples$run

groups3<-rung$samples$environment

groups4<-rung$samples$tree_id

## Marti Anderson's PERMDISP2 procedure for the analysis of multivariate homogeneity of group dispersions (variances).

mod <- vegan::betadisper(dis, groups1)

anova(mod)

vegan::permutest(mod, pairwise = TRUE, permutations = 999)

mod <- vegan::betadisper(dis, groups2)

anova(mod)

vegan::permutest(mod, pairwise = TRUE, permutations = 999)

mod <- vegan::betadisper(dis, groups3)

anova(mod)

vegan::permutest(mod, pairwise = TRUE, permutations = 999)

mod <- vegan::betadisper(dis, groups4)

anova(mod)

vegan::permutest(mod, pairwise = TRUE, permutations = 999)

rm(mod,groups1,groups2,groups3,groups4,matrix,dis)

## PERMANOVA: after moving factors around, this seems the right model

perform_adonis(rung,c("type","run","environment","tree_id","days_since_leaf_emergence","run:type","environment:type","type:days_since_leaf_emergence"),permutations=10000)

```

### Community dynamics on leaves

# Distance matrix

```{r}

# only need data points one week apart:

runl<-run%>%

filter_samples(type=="Leaves")%>%

filter_samples(run=="Antwerp")

runw<-run%>%

filter_samples(type%in%c("Trunk","Branch"))%>%

filter_samples(days_since_leaf_emergence!=3)%>%

filter_samples(run=="Antwerp")

runf<-merge_tidyamplicons(runl,runw)

runf$samples$tree_id<-as.factor(runf$samples$tree_id)

dist<-get_betas(runf, method = "bray", unique = FALSE)

(dist[1:5,])

```

# Distances between (previous and) next time point for each plant, Figure 2A

```{r}

c<-c()

for (i in seq(1,nrow(dist),by=1)){

if (dist[i,"tree_id_1"]==dist[i,"tree_id_2"]){

if (dist[i,"type_1"]==dist[i,"type_2"]){

d1 <- dist[i,"timepoint_1"]

d2 <- dist[i,"timepoint_2"]

if (d1+1==d2 ) c<-append(c,i)} #d1-1==d2 ||

}

}

dist1<-dist[c,]

dist1$tree_id_1<-as.factor(dist1$tree_id_1)

ggplot(dist1, aes(x=days_since_leaf_emergence_2, y=beta)) +

geom_point(size=2.5, aes(colour=tree_id_1,shape=environment_1)) +

geom_errorbar(stat = "summary", fun.y ="mean", width=1.5, aes(ymax=..y.., ymin=..y.., group=days_since_leaf_emergence_2))+

scale_color_manual(values=c("#650d1b","#9b3d12","#ae8e1c","#3c1518"))+

scale_shape(name="City")+

facet_grid(~type_1)+

xlab("Time (days)") +

ylab("Dissimilarity with previous time point") +

theme_bw()+

theme(

panel.grid=element_line(colour="black"),

axis.title.y = element_text(size = 12,margin=margin(0,15,0,0)),

axis.title.x = element_text(size = 12,margin=margin(15,0,0,0)),

axis.text.x = element_text(size = 12,colour="black",angle=45,hjust=1),

axis.text.y = element_text(size = 12,colour="black"),

axis.ticks.x = element_line(colour = "black"),

axis.ticks.y = element_line(colour = "black"))+

theme(axis.line = element_line(colour = "black"),

panel.grid.major = element_blank(),

panel.grid.minor = element_blank(),

panel.background = element_blank(),

legend.key = element_blank(),

strip.background = element_rect(fill = "white",

colour = "black",

size = 0.2),

strip.text.x = element_text(size=10,margin=margin(7,0,7,0)))+

theme(legend.position="none")+

theme(axis.title.y = element_text(hjust=0.7))

```

# Let's test!

```{r,fig.height = 5}

dist1l<-filter(dist1,type_1=="Leaves")

dist1b<-filter(dist1,type_1=="Branch")

dist1t<-filter(dist1,type_1=="Trunk")

cor.test(dist1$timepoint_2,dist1$beta,method="kendall")

cor.test(dist1l$timepoint_2,dist1l$beta,method="kendall")

cor.test(dist1t$timepoint_2,dist1t$beta,method="kendall")

cor.test(dist1b$timepoint_2,dist1b$beta,method="kendall")

```

# Distances within timepoint, Figure 2B

Distances between types: no trends

```{r}

c<-c()

for (i in seq(1,nrow(dist),by=1)){

if (dist[i,"timepoint_1"]==dist[i,"timepoint_2"]){

if (dist[i,"type_1"]==dist[i,"type_2"]){

# if (dist[i,"environment_1"]==dist[i,"environment_2"]){

t1 <- dist[i,"tree_id_1"]

t2 <- dist[i,"tree_id_2"]

if (t1!=t2 ) c<-append(c,i)}

}

}

dist2<-dist[c,]

dist2$tree_id_1<-as.factor(dist2$tree_id_1)

dist2<-filter(dist2,days_since_leaf_emergence_1>-1)

ggplot(dist2, aes(x=days_since_leaf_emergence_2, y=beta)) +

geom_point(size=2, aes(colour=type_1)) +

geom_errorbar(stat = "summary", fun.y ="mean", width=1.5, aes(ymax=..y.., ymin=..y.., group=interaction(timepoint_1,type_1),colour=tree_id_1))+

#geom_text(label=dist2$tree_id_1,size=2,vjust=1.5)+

scale_colour_manual(values=c("darkorange3","chartreuse3","darkorange4"))+

scale_shape(name="City")+

facet_grid(~type_1)+

xlab("Time (days)") +

ylab("Dissimilarity with other trees") +

theme_bw()+

theme(

panel.grid=element_line(colour="black"),

axis.title.y = element_text(size = 12,margin=margin(0,15,0,0)),

axis.title.x = element_text(size = 12,margin=margin(15,0,0,0)),

axis.text.x = element_text(size = 12,colour="black",angle=45,hjust=1),

axis.text.y = element_text(size = 12,colour="black"),

axis.ticks.x = element_line(colour = "black"),

axis.ticks.y = element_line(colour = "black"))+

theme(axis.line = element_line(colour = "black"),

panel.grid.major = element_blank(),

panel.grid.minor = element_blank(),

panel.background = element_blank(),

legend.key = element_blank(),

strip.background = element_rect(fill = "white",

colour = "black",

size = 0.2),

strip.text.x = element_text(size=10,margin=margin(7,0,7,0)))+

theme(legend.position="none")

```

# Let's test!

```{r,fig.height = 5}

dist2l<-filter(dist2,type_1=="Leaves")

dist2b<-filter(dist2,type_1=="Branch")

dist2t<-filter(dist2,type_1=="Trunk")

cor.test(dist2$timepoint_2,dist2$beta,method="kendall")

cor.test(dist2l$timepoint_2,dist2l$beta,method="kendall")

cor.test(dist2t$timepoint_2,dist2t$beta,method="kendall")

cor.test(dist2b$timepoint_2,dist2b$beta,method="kendall")

rm(dist,dist2,dist2l,dist2b,dist2t,dist1,dist1l,dist1b,dist1t,runw,runl,runf,d1,d2,t1,t2,c,i)

```

### Co-occurrence clusters

#libraries and functions

```{r,fig.height = 5}

library(factoextra) # need R 3.6

add_tax_abundance <- function(ta) {

# make table with taxon and total abundance

tot_abundances <- ta$abundances %>%

group_by(taxon_id) %>%

summarize(tot_abundance = sum(abundance))

# add total abundance to taxon table

ta$taxa <- left_join(ta$taxa, tot_abundances)

# return ta object

ta

}

as_abundances_matrix2 <- function(abundances, value = abundance) {

if (

! is.data.frame(abundances) |

is.null(abundances$taxon_name) |

is.null(abundances$sample_id)

) stop("need abundances in right format")

value <- enquo(value)

abundances_wide <- abundances %>%

select(sample_id, taxon_name, !! value) %>%

spread(key = taxon_name, value = !! value, fill = 0)

abundances_wide %>%

select(- sample_id) %>%

as.matrix() %>%

`row.names<-`(abundances_wide$sample_id)

}

'%!in%' <- function(x,y)!('%in%'(x,y))

```

# Repeated hierarchical clustering

# Leaves

```{r,fig.height = 5}

# Select samples

runl<-run%>%

filter_samples(run=="Antwerp")%>%

filter_samples(type=="Leaves")%>%

add_lib_size()%>%

filter_samples(lib_size>911)%>%

add_total_rel_abundance()%>%

add_tax_abundance()

# List of all leaf taxa:

runleaf<-runl%>%

add_rel_abundance()

leaftax<-select(runleaf$taxa,taxon_name,total_rel_abundance)

rm(runleaf)

r<-runl%>%

add_rel_abundance()%>%

mutate_abundances(abundance=rel_abundance)%>%

add_total_rel_abundance()

ggplot(runl$taxa)+

geom_freqpoly(aes(total_rel_abundance*91200))+

geom_point(aes(x=25, y=35))+

xlim(c(0,100))+

xlab("Total abundance of ASV in dataset")+

ylab("Number of different ASVs")+

theme_bw()

runl<-runl%>%

select_taxa(taxon_id,taxon_name)

clusseed<-c()

optclust<-c()

seedlate<-sample(c("Skermanella 1","Pantoea"),1000,replace=TRUE)

for (i in seq(1,1000,1)){

set.seed(1100+i)

runr<-runl%>%

rarefy(912)

runclust<-runr%>%

add_rel_abundance()%>%

add_tax_abundance()

# Call ASVs that have less than 25 reads in total "Other"

runclust$taxa<-runclust$taxa%>%

mutate(taxon_name = ifelse(tot_abundance<25, "Other", taxon_name))

runclust<-runclust%>%

select_taxa(taxon_id,taxon_name)%>%

aggregate_taxa()%>%

add_tax_abundance()

# Scale ASV abundances using their "total occurrence"

runclust_scaled<-runclust

runclust_scaled$abundances<-left_join(runclust_scaled$abundances,runclust_scaled$taxa)

runclust_scaled<-runclust_scaled%>%

mutate_abundances(abundance=abundance/tot_abundance)

table<-(as.data.frame(as_abundances_matrix2(runclust_scaled$abundances)))

tab<-t(table)

# Remove outliers

runclust_scaled<-runclust_scaled%>%

filter_taxa(! taxon_name %in% c("Other","Hymenobacter 53","Actimicrobium 1"))%>% # removing "Other" and ASVs that are often outliers

process_taxon_selection()

table<-(as.data.frame(as_abundances_matrix2(runclust_scaled$abundances)))

tab<-t(table)

clustable<-table

n_clust<-fviz_nbclust(clustable, hcut, method = "silhouette", k.max = 16)

n_clust<-n_clust$data

max_cluster<-as.numeric(n_clust$clusters[which.max(n_clust$y)])

optclust<-append(optclust,max_cluster)

dis<-vegan::vegdist(tab, "euclidean")

clus<-hclust(dis, method = "ward.D2")

clu<-as.matrix(cutree(clus, k = 4))

clu<-cbind(clu,rownames(clu))

clu<-as_tibble(clu)

colnames(clu)<-c(paste0("cluster",4),"taxon_name")

runclust$taxa<-left_join(runclust$taxa,clu)

sphingo<-runclust$taxa%>%

filter(taxon_name=="Sphingomonas 1")

sphingo<-sphingo$cluster4

if (sphingo==1){

runclust$taxa<-runclust$taxa%>%

mutate(cluster4=ifelse(cluster4==1,"General cluster",cluster4))}

if (sphingo==2){

runclust$taxa<-runclust$taxa%>%

mutate(cluster4=ifelse(cluster4==2,"General cluster",cluster4))}

if (sphingo==3){

runclust$taxa<-runclust$taxa%>%

mutate(cluster4=ifelse(cluster4==3,"General cluster",cluster4))}

if (sphingo==4){

runclust$taxa<-runclust$taxa%>%

mutate(cluster4=ifelse(cluster4==4,"General cluster",cluster4))}

st2<-runclust$taxa%>%

filter(taxon_name==seedlate[i])

st2<-st2$cluster4

clusseed<-append(clusseed,st2=="General cluster")

if (st2==1){

runclust$taxa<-runclust$taxa%>%

mutate(cluster4=ifelse(cluster4==1,"Late cluster",cluster4))}

if (st2==2){

runclust$taxa<-runclust$taxa%>%

mutate(cluster4=ifelse(cluster4==2,"Late cluster",cluster4))}

if (st2==3){

runclust$taxa<-runclust$taxa%>%

mutate(cluster4=ifelse(cluster4==3,"Late cluster",cluster4))}

if (st2==4){

runclust$taxa<-runclust$taxa%>%

mutate(cluster4=ifelse(cluster4==4,"Late cluster",cluster4))}

ln<-c(1,2,3,4)

leftover_no<-ln[ln %!in% c(sphingo,st2)]

runclust$taxa<-runclust$taxa%>%

mutate(cluster4=ifelse(cluster4==leftover_no[1],"Mid cluster",cluster4))

runclust$taxa<-runclust$taxa%>%

mutate(cluster4=ifelse(cluster4==leftover_no[2],"Early cluster",cluster4))

runclust$taxa$cluster4<-factor(runclust$taxa$cluster4, levels = c("General cluster","Early cluster" ,"Mid cluster","Late cluster"))

t<-runclust$taxa%>%

select(taxon_name,cluster4)

colnames(t)[2]<-c(paste("seed",(1100+i),sep=""))

leaftax<-left_join(leaftax,t)

}

hist(optclust)

TRUE%in%clusseed #needs to be false to verify good choice of late cluster seed

lt<-leaftax%>%

gather("seed","cluster",-taxon_name,-total_rel_abundance)%>%

replace_na(list(cluster="none"))%>%

group_by(taxon_name)%>%

mutate(pct_gen=mean(cluster=="General cluster"))%>%

mutate(pct_mid=mean(cluster=="Mid cluster"))%>%

mutate(pct_lat=mean(cluster=="Late cluster"))%>%

mutate(pct_ear=mean(cluster=="Early cluster"))%>%

select(-seed,-cluster)%>%

unique()%>%

mutate(cluster4=0)

for (row in 1:nrow(lt)) {

gen <- lt[row, "pct_gen"]

mid <- lt[row, "pct_mid"]

lat <- lt[row, "pct_lat"]

ear <- lt[row, "pct_ear"]

if(gen > mid & gen > lat & gen > ear) {

lt[row,"cluster4"]<-1

lt[row,"support"]<-gen

}

if(mid > gen & mid > lat & mid > ear) {

lt[row,"cluster4"]<-2

lt[row,"support"]<-mid

}

if(lat > gen & lat > mid & lat > ear) {

lt[row,"cluster4"]<-3

lt[row,"support"]<-lat

}

if(ear > gen & ear > mid & ear > lat) {

lt[row,"cluster4"]<-4

lt[row,"support"]<-ear

}

}

gentax<-lt%>%

ungroup()%>%

filter(cluster4==1)%>%

arrange(desc(total_rel_abundance))%>%

select(taxon_name,support,total_rel_abundance)

midtax<-lt%>%

ungroup()%>%

filter(cluster4==2)%>%

arrange(desc(total_rel_abundance))%>%

select(taxon_name,support,total_rel_abundance)

lattax<-lt%>%

ungroup()%>%

filter(cluster4==3)%>%

arrange(desc(total_rel_abundance))%>%

select(taxon_name,support,total_rel_abundance)

eartax<-lt%>%

ungroup()%>%

filter(cluster4==4)%>%

arrange(desc(total_rel_abundance))%>%

select(taxon_name,support,total_rel_abundance)

table<-rbind(gentax,eartax,midtax,lattax)

write.table(table,"allclustertax.txt",sep="\t")

taxclu<-lt%>%

select(taxon_name,cluster4)

taxclust<-taxclu

runcluster<-runl%>%

select_samples(sample_id,type,run, timepoint, days_since_leaf_emergence, tree_id,environment)%>%

add_rel_abundance()

runcluster$taxa<-left_join(runcluster$taxa,taxclu)%>%

filter(cluster4>0)%>%

drop_na(cluster4)

alldata<-left_join(runcluster$abundances,runcluster$taxa)%>%

drop_na(cluster4)

```

# Figure 3

```{r,fig.height = 5}

c4data<-alldata%>%

group_by(sample_id,cluster4)%>%

summarize(cluster_abundance = sum(rel_abundance))

sample_id<-rep(runcluster$samples$sample_id,4)

l<-length(sample_id)

cluster4<-c(rep(1,l/4),rep(2,l/4),rep(3,l/4),rep(4,l/4))

complete<-as_tibble(cbind(sample_id,cluster4))

complete$cluster4<- as.integer(complete$cluster4)

c4data<-left_join(complete,c4data, by = c("cluster4" = "cluster4", "sample_id" = "sample_id"))

c4data<-c4data%>%

replace_na(list(cluster_abundance=0))%>%

left_join(runcluster$samples)

c4data<-c4data%>%

mutate(cluster4=ifelse(cluster4==2,"Mid" ,cluster4))%>%

mutate(cluster4=ifelse(cluster4==1,"General",cluster4))%>%

mutate(cluster4=ifelse(cluster4==3,"Late",cluster4))%>%

mutate(cluster4=ifelse(cluster4==4,"Early",cluster4))

c4data$cluster4<-factor(c4data$cluster4, levels = c("General","Early" ,"Mid","Late"))

c4data$tree_id<-as.character(c4data$tree_id)

ggplot(c4data,aes(x=days_since_leaf_emergence,y=cluster_abundance))+

geom_boxplot(width=3.5,alpha=0.5,color='grey',aes(group=interaction(cluster4, days_since_leaf_emergence)))+

geom_point(size=2,alpha=0.8, aes(shape=environment,color=tree_id))+

#geom_text(label=c4data$tree_id,size=2,vjust=1.5)+

scale_color_manual(values=c("#153940","#8A8A3A","#577A40","#C49443"),guide=FALSE)+

facet_wrap(~cluster4,ncol=4)+

ylab("Relative abundance of cluster")+

xlab("Days since leaf emergence")+

scale_y_continuous(limits=c(0,1.01))+

labs(shape="Land use")+

theme_bw()+

theme(

panel.grid=element_line(colour="black"),

axis.title.y = element_text(size = 10,margin=margin(0,15,0,0)),

axis.title.x = element_text(size = 10,margin=margin(15,0,0,0)),

axis.text.x = element_text(size = 10,colour="black",angle=45,hjust=1),

axis.text.y = element_text(size = 10,colour="black"),

axis.ticks.x = element_line(colour = "black"),

axis.ticks.y = element_line(colour = "black"))+

theme(axis.line = element_line(colour = "black"),

panel.grid.major = element_blank(),

panel.grid.minor = element_blank(),

panel.background = element_blank(),

legend.key = element_blank(),

strip.background = element_rect(fill = "white",

colour = "black",

size = 0.2),

strip.text.x = element_text(size=10,margin=margin(7,0,7,0)))

```

# Figure S3

```{r,fig.height = 5}

runclu<-run%>%

filter_samples(type=="Leaves")%>%

filter_samples(lib_size>911)%>%

add_rel_abundance()

runclu$samples<-runclu$samples%>%

unite("Location",run,environment,sep=" ")

runclu$taxa<-left_join(runclu$taxa,taxclust)%>%

filter(cluster4>0)%>%

drop_na(cluster4)

alldata<-left_join(runclu$abundances,runclu$taxa)%>%

drop_na(cluster4)

c4data<-alldata%>%

group_by(sample_id,cluster4)%>%

summarize(cluster_abundance = sum(rel_abundance))

sample_id<-rep(runclu$samples$sample_id,4)

l<-length(sample_id)

cluster4<-c(rep(1,l/4),rep(2,l/4),rep(3,l/4),rep(4,l/4))

complete<-as_tibble(cbind(sample_id,cluster4))

complete$cluster4<- as.integer(complete$cluster4)

c4data<-left_join(complete,c4data, by = c("cluster4" = "cluster4", "sample_id" = "sample_id"))

c4data<-c4data%>%

replace_na(list(cluster_abundance=0))%>%

left_join(runclu$samples)

c4data<-c4data%>%

mutate(cluster4=ifelse(cluster4==2,"Mid" ,cluster4))%>%

mutate(cluster4=ifelse(cluster4==1,"General",cluster4))%>%

mutate(cluster4=ifelse(cluster4==3,"Late",cluster4))%>%

mutate(cluster4=ifelse(cluster4==4,"Early",cluster4))

c4data$cluster4<-factor(c4data$cluster4, levels = c("General","Early" ,"Mid","Late"))

c4data$cluster_abundance<-c4data$cluster_abundance+0.001

ggplot(c4data,aes(x=days_since_leaf_emergence,y=cluster_abundance))+

geom_boxplot(width=3.5,alpha=0.5,color='grey',aes(group=interaction(cluster4, days_since_leaf_emergence)))+

geom_point(size=2,alpha=0.8, aes(shape=Location))+

scale_shape_manual(values=c(0,1,15,16))+

facet_wrap(~cluster4,ncol=2)+

ylab("Relative abundance of cluster")+

xlab("Days since leaf emergence")+

scale_y_log10(limits=c(0.001,1.01))+

labs(shape="Land use")+

theme_bw()+

theme(

panel.grid=element_line(colour="black"),

axis.title.y = element_text(size = 10,margin=margin(0,15,0,0)),

axis.title.x = element_text(size = 10,margin=margin(15,0,0,0)),

axis.text.x = element_text(size = 10,colour="black",angle=45,hjust=1),

axis.text.y = element_text(size = 10,colour="black"),

axis.ticks.x = element_line(colour = "black"),

axis.ticks.y = element_line(colour = "black"))+

theme(axis.line = element_line(colour = "black"),

panel.grid.major = element_blank(),

panel.grid.minor = element_blank(),

panel.background = element_blank(),

# legend.key = element_blank(),

strip.background = element_rect(fill = "white",

colour = "black",

size = 0.2),

strip.text.x = element_text(size=10,margin=margin(7,0,7,0)))

rm(ear,gen,mid,lat,runclust,runclust_scaled,runcluster,table,clu,clus,c4data,clustable,complete,taxclu,n_clust,row,optclust,seedlate,sphingo,st2,clusseed,cluster4,max_cluster,sample_id,lt,alldata,runl,runr,t,tab,i,l,leftover_no,ln)

```

# Repeated hierarchical clustering: BRANCH

```{r,fig.height = 5}

runl<-run%>%

filter_samples(run=="Antwerp")%>%

filter_samples(type=="Branch")%>%

add_lib_size()%>%

filter_samples(lib_size>911)%>%

add_total_rel_abundance()%>%

add_tax_abundance()

# List of all branch taxa:

runleaf<-runl%>%

add_rel_abundance()

branchtax<-select(runleaf$taxa,taxon_name,total_rel_abundance)

rm(runleaf)

r<-runl%>%

add_rel_abundance()%>%

mutate_abundances(abundance=rel_abundance)%>%

add_total_rel_abundance()

ggplot(runl$taxa)+

geom_freqpoly(aes(total_rel_abundance*91200))+

xlim(c(0,25))+

xlab("Total abundance of ASV in dataset")+

ylab("Number of different ASVs")+

theme_bw()

runl<-runl%>%

select_taxa(taxon_id,taxon_name)

clusseed<-c()

optclust<-c()

for (i in seq(1,100,1)){

set.seed(1100+i)

runr<-runl%>%

rarefy(912)

runclust<-runr%>%

add_rel_abundance()%>%

add_tax_abundance()

# Call ASVs that have less than 25 reads in total "Other"

runclust$taxa<-runclust$taxa%>%

mutate(taxon_name = ifelse(tot_abundance<25, "Other", taxon_name))

runclust<-runclust%>%

select_taxa(taxon_id,taxon_name)%>%

aggregate_taxa()%>%

add_tax_abundance()

# Scale ASV abundances using their "total occurrence"

runclust_scaled<-runclust

runclust_scaled$abundances<-left_join(runclust_scaled$abundances,runclust_scaled$taxa)

runclust_scaled<-runclust_scaled%>%

mutate_abundances(abundance=abundance/tot_abundance)

table<-(as.data.frame(as_abundances_matrix2(runclust_scaled$abundances)))

tab<-t(table)

# Remove outliers

runclust_scaled<-runclust_scaled%>%

filter_taxa(! taxon_name %in% c("Other"))%>% # removing "Other"

process_taxon_selection()

table<-(as.data.frame(as_abundances_matrix2(runclust_scaled$abundances)))

tab<-t(table)

clustable<-table

n_clust<-fviz_nbclust(clustable, hcut, method = "silhouette", k.max = 16)

n_clust<-n_clust$data

max_cluster<-as.numeric(n_clust$clusters[which.max(n_clust$y)])

optclust<-append(optclust,max_cluster)

dis<-vegan::vegdist(tab, "euclidean")

clus<-hclust(dis, method = "ward.D2")

clu<-as.matrix(cutree(clus, k = 2))

clu<-cbind(clu,rownames(clu))

clu<-as_tibble(clu)

colnames(clu)<-c(paste0("cluster",4),"taxon_name")

runclust$taxa<-left_join(runclust$taxa,clu)

t<-runclust$taxa%>%

select(taxon_name,cluster4)

colnames(t)[2]<-c(paste("seed",(1100+i),sep=""))

branchtax<-left_join(branchtax,t)

}

#View(optclust)

hist(optclust, breaks=16)

lt<-branchtax%>%

gather("seed","cluster",-taxon_name,-total_rel_abundance)%>%

replace_na(list(cluster="none"))%>%

group_by(taxon_name)%>%

mutate(pct_1=mean(cluster==1))%>%

mutate(pct_2=mean(cluster==2))%>%

select(-seed,-cluster)%>%

unique()%>%

mutate(cluster4=0)

for (row in 1:nrow(lt)) {

gen <- lt[row, "pct_1"]

mid <- lt[row, "pct_2"]

if(gen > mid ) {

lt[row,"cluster4"]<-1

lt[row,"support"]<-gen

}

if(mid > gen ) {

lt[row,"cluster4"]<-2

lt[row,"support"]<-mid

}

}

firtax<-lt%>%

ungroup()%>%

filter(cluster4==1)%>%

arrange(desc(total_rel_abundance))%>%

select(taxon_name,support,total_rel_abundance)

sectax<-lt%>%

ungroup()%>%

filter(cluster4==2)%>%

arrange(desc(total_rel_abundance))%>%

select(taxon_name,support,total_rel_abundance)

intersect(firtax$taxon_name,eartax$taxon_name)

intersect(firtax$taxon_name,midtax$taxon_name)

intersect(firtax$taxon_name,lattax$taxon_name)

length(intersect(firtax$taxon_name,gentax$taxon_name))/nrow(firtax)

length(intersect(firtax$taxon_name,leaftax$taxon_name))/nrow(firtax)

intersect(sectax$taxon_name,eartax$taxon_name)

intersect(sectax$taxon_name,midtax$taxon_name)

intersect(sectax$taxon_name,lattax$taxon_name)

intersect(sectax$taxon_name,gentax$taxon_name)

length(intersect(sectax$taxon_name,gentax$taxon_name))/nrow(sectax)

length(intersect(sectax$taxon_name,leaftax$taxon_name))/nrow(sectax)

taxclu<-lt%>%

select(taxon_name,cluster4)

runcluster<-runl%>%

select_samples(sample_id,type,run, timepoint, days_since_leaf_emergence, tree_id,environment)%>%

#select_taxa(-sequence)%>%

add_rel_abundance()

runcluster$taxa<-left_join(runcluster$taxa,taxclu)%>%

filter(cluster4>0)%>%

drop_na(cluster4)

alldata<-left_join(runcluster$abundances,runcluster$taxa)%>%

drop_na(cluster4)

c4data<-alldata%>%

group_by(sample_id,cluster4)%>%

summarize(cluster_abundance = sum(rel_abundance))

sample_id<-rep(runcluster$samples$sample_id,2)

l<-length(sample_id)

cluster4<-c(rep(1,l/2),rep(2,l/2))

complete<-as_tibble(cbind(sample_id,cluster4))

complete$cluster4<- as.integer(complete$cluster4)

c4data<-left_join(complete,c4data, by = c("cluster4" = "cluster4", "sample_id" = "sample_id"))

c4data<-c4data%>%

replace_na(list(cluster_abundance=0))%>%

left_join(runcluster$samples)

c4data$tree_id<-as.character(c4data$tree_id)

ggplot(c4data,aes(x=days_since_leaf_emergence,y=cluster_abundance))+

geom_boxplot(width=3.5,alpha=0.5,color='grey',aes(group=interaction(cluster4, days_since_leaf_emergence)))+

geom_point(size=2,alpha=0.8, aes(shape=environment,color=tree_id))+

#geom_text(label=c4data$tree_id,size=2,vjust=1.5)+

scale_color_manual(values=c("#153940","#8A8A3A","#577A40","#C49443"),guide=FALSE)+

facet_wrap(~cluster4,ncol=2)+

ylab("Relative abundance of cluster")+

xlab("Days since leaf emergence")+

#coord_cartesian(ylim=c(0,1.01))+

scale_y_continuous(limits=c(0,1.01))+ #trans='log2',

labs(shape="Land use")+

theme_bw()

ggplot(c4data,aes(x=environment,y=cluster_abundance))+

geom_boxplot(alpha=0.5,color='grey',aes(group=interaction(environment, cluster4)))+

geom_point(size=2,alpha=0.8, aes(shape=environment,color=tree_id))+

#geom_text(label=c4data$tree_id,size=2,vjust=1.5)+

scale_color_manual(values=c("#153940","#8A8A3A","#577A40","#C49443"),guide=FALSE)+

facet_wrap(~cluster4,ncol=4)+

ylab("Relative abundance of cluster")+

xlab(" ")+

#coord_cartesian(ylim=c(0,1.01))+

scale_y_continuous(limits=c(0,1.01))+ #trans='log2',

labs(shape="Land use")+

theme_bw()

c4datap<-c4data%>%

#filter(environment=="park")%>%

filter(cluster4==2)

cor.test(c4datap$cluster_abundance,c4datap$days_since_leaf_emergence,method="kendall")

```

# Branch with 5 clusters (not in manuscript)

nothing more than what's happening with 2 clusters

```{r,fig.height = 5}

clusseed<-c()

optclust<-c()

for (i in seq(1,100,1)){

set.seed(1100+i)

runr<-runl%>%

rarefy(912)

runclust<-runr%>%

add_rel_abundance()%>%

add_tax_abundance()

# Call ASVs that have less than 25 reads in total "Other"

runclust$taxa<-runclust$taxa%>%

mutate(taxon_name = ifelse(tot_abundance<25, "Other", taxon_name))

runclust<-runclust%>%

select_taxa(taxon_id,taxon_name)%>%

aggregate_taxa()%>%

add_tax_abundance()

# Scale ASV abundances using their "total occurrence"

runclust_scaled<-runclust

runclust_scaled$abundances<-left_join(runclust_scaled$abundances,runclust_scaled$taxa)

runclust_scaled<-runclust_scaled%>%

mutate_abundances(abundance=abundance/tot_abundance)

table<-(as.data.frame(as_abundances_matrix2(runclust_scaled$abundances)))

tab<-t(table)

# Remove outliers

runclust_scaled<-runclust_scaled%>%

filter_taxa(! taxon_name %in% c("Other"))%>% # removing "Other"

process_taxon_selection()

table<-(as.data.frame(as_abundances_matrix2(runclust_scaled$abundances)))

tab<-t(table)

clustable<-table

n_clust<-fviz_nbclust(clustable, hcut, method = "silhouette", k.max = 16)

n_clust<-n_clust$data

max_cluster<-as.numeric(n_clust$clusters[which.max(n_clust$y)])

optclust<-append(optclust,max_cluster)

dis<-vegan::vegdist(tab, "euclidean")

clus<-hclust(dis, method = "ward.D2")

clu<-as.matrix(cutree(clus, k = 5))

clu<-cbind(clu,rownames(clu))

clu<-as_tibble(clu)

colnames(clu)<-c(paste0("cluster",4),"taxon_name")

runclust$taxa<-left_join(runclust$taxa,clu)

t<-runclust$taxa%>%

select(taxon_name,cluster4)

colnames(t)[2]<-c(paste("seed",(1100+i),sep=""))

branchtax<-left_join(branchtax,t)

}

#View(optclust)

hist(optclust, breaks=16)

lt<-branchtax%>%

gather("seed","cluster",-taxon_name,-total_rel_abundance)%>%

replace_na(list(cluster="none"))%>%

group_by(taxon_name)%>%

mutate(pct_1=mean(cluster==1))%>%

mutate(pct_2=mean(cluster==2))%>%

mutate(pct_3=mean(cluster==3))%>%

mutate(pct_4=mean(cluster==4))%>%

mutate(pct_5=mean(cluster==5))%>%

select(-seed,-cluster)%>%

unique()%>%

mutate(cluster4=0)

for (row in 1:nrow(lt)) {

on <- lt[row, "pct_1"]

tw <- lt[row, "pct_2"]

th <- lt[row, "pct_3"]

fo <- lt[row, "pct_4"]

fi <- lt[row, "pct_5"]

if(on > tw & on > th & on > fo & on > fi ) {

lt[row,"cluster4"]<-1

lt[row,"support"]<-on

}

if(tw > on & tw > th & tw > fo & tw > fi ) {

lt[row,"cluster4"]<-2

lt[row,"support"]<-tw

}

if(th > on & th > tw & th > fo & th > fi ) {

lt[row,"cluster4"]<-3

lt[row,"support"]<-th

}

if(fo > on & fo > tw & fo > th & fo > fi ) {

lt[row,"cluster4"]<-4

lt[row,"support"]<-fo

}

if(fi > on & fi > tw & fi > th & fi > fo ) {

lt[row,"cluster4"]<-5

lt[row,"support"]<-fi

}

}

taxclu<-lt%>%

select(taxon_name,cluster4)

runcluster<-runl%>%

select_samples(sample_id,type,run, timepoint, days_since_leaf_emergence, tree_id,environment)%>%

#select_taxa(-sequence)%>%

add_rel_abundance()

runcluster$taxa<-left_join(runcluster$taxa,taxclu)%>%

filter(cluster4>0)%>%

drop_na(cluster4)

alldata<-left_join(runcluster$abundances,runcluster$taxa)%>%

drop_na(cluster4)

c4data<-alldata%>%

group_by(sample_id,cluster4)%>%

summarize(cluster_abundance = sum(rel_abundance))

sample_id<-rep(runcluster$samples$sample_id,5)

l<-length(sample_id)

cluster4<-c(rep(1,l/5),rep(2,l/5),rep(3,l/5),rep(4,l/5),rep(5,l/5))

complete<-as_tibble(cbind(sample_id,cluster4))

complete$cluster4<- as.integer(complete$cluster4)

c4data<-left_join(complete,c4data, by = c("cluster4" = "cluster4", "sample_id" = "sample_id"))

c4data<-c4data%>%

replace_na(list(cluster_abundance=0))%>%

left_join(runcluster$samples)

c4data$tree_id<-as.character(c4data$tree_id)

ggplot(c4data,aes(x=days_since_leaf_emergence,y=cluster_abundance))+

geom_boxplot(width=3.5,alpha=0.5,color='grey',aes(group=interaction(cluster4, days_since_leaf_emergence)))+

geom_point(size=2,alpha=0.8, aes(shape=environment,color=tree_id))+

#geom_text(label=c4data$tree_id,size=2,vjust=1.5)+

scale_color_manual(values=c("#153940","#8A8A3A","#577A40","#C49443"),guide=FALSE)+

facet_wrap(~cluster4,ncol=3)+

ylab("Relative abundance of cluster")+

xlab("Days since leaf emergence")+

#scale_y_continuous(trans='log2',limits=c(0.001,1.01))+

labs(shape="Land use")+

theme_bw()

ggplot(c4data,aes(x=tree_id,y=cluster_abundance))+

geom_boxplot(alpha=0.5,color='grey',aes(group=interaction(environment, cluster4)))+

geom_point(size=2,alpha=0.8, aes(shape=environment,color=tree_id))+

#geom_text(label=c4data$tree_id,size=2,vjust=1.5)+

scale_color_manual(values=c("#153940","#8A8A3A","#577A40","#C49443"),guide=FALSE)+

facet_wrap(~cluster4,ncol=4)+

ylab("Relative abundance of cluster")+

xlab(" ")+

#scale_y_continuous(limits=c(0,1.01))+

labs(shape="Land use")+

theme_bw()

c4datap<-c4data%>%

#filter(environment=="park")%>%

filter(cluster4==2)

cor.test(c4datap$cluster_abundance,c4datap$days_since_leaf_emergence,method="kendall")

```

# Repeated hierarchical clustering: TRUNK

```{r,fig.height = 5}

runl<-run%>%

filter_samples(run=="Antwerp")%>%

filter_samples(type=="Trunk")%>%

add_lib_size()%>%

filter_samples(lib_size>911)%>%

add_total_rel_abundance()%>%

add_tax_abundance()

# List of all trunk taxa:

runleaf<-runl%>%

add_rel_abundance()

trunktax<-select(runleaf$taxa,taxon_name,total_rel_abundance)

rm(runleaf)

r<-runl%>%

add_rel_abundance()%>%

mutate_abundances(abundance=rel_abundance)%>%

add_total_rel_abundance()

ggplot(runl$taxa)+

geom_freqpoly(aes(total_rel_abundance*91200))+

xlim(c(0,25))+

xlab("Total abundance of ASV in dataset")+

ylab("Number of different ASVs")+

theme_bw()

runl<-runl%>%

select_taxa(taxon_id,taxon_name)

clusseed<-c()

optclust<-c()

for (i in seq(1,100,1)){

set.seed(1100+i)

runr<-runl%>%

rarefy(912)

runclust<-runr%>%

add_rel_abundance()%>%

add_tax_abundance()

# Call ASVs that have less than 25 reads in total "Other"

runclust$taxa<-runclust$taxa%>%

mutate(taxon_name = ifelse(tot_abundance<25, "Other", taxon_name))

runclust<-runclust%>%

select_taxa(taxon_id,taxon_name)%>%

aggregate_taxa()%>%

add_tax_abundance()

# Scale ASV abundances using their "total occurrence"

runclust_scaled<-runclust

runclust_scaled$abundances<-left_join(runclust_scaled$abundances,runclust_scaled$taxa)

runclust_scaled<-runclust_scaled%>%

mutate_abundances(abundance=abundance/tot_abundance)

table<-(as.data.frame(as_abundances_matrix2(runclust_scaled$abundances)))

tab<-t(table)

# Remove outliers

runclust_scaled<-runclust_scaled%>%

filter_taxa(! taxon_name %in% c("Other"))%>% # removing "Other"

process_taxon_selection()

table<-(as.data.frame(as_abundances_matrix2(runclust_scaled$abundances)))

tab<-t(table)

clustable<-table

n_clust<-fviz_nbclust(clustable, hcut, method = "silhouette", k.max = 16)

n_clust<-n_clust$data

max_cluster<-as.numeric(n_clust$clusters[which.max(n_clust$y)])

optclust<-append(optclust,max_cluster)

dis<-vegan::vegdist(tab, "euclidean")

clus<-hclust(dis, method = "ward.D2")

clu<-as.matrix(cutree(clus, k = 3))

clu<-cbind(clu,rownames(clu))

clu<-as_tibble(clu)

colnames(clu)<-c(paste0("cluster",4),"taxon_name")

runclust$taxa<-left_join(runclust$taxa,clu)

se1<-runclust$taxa%>%

filter(taxon_name=="Sphingomonas 2")

se1<-se1$cluster4

if (se1==1){

runclust$taxa<-runclust$taxa%>%

mutate(cluster4=ifelse(cluster4==1,"fi",cluster4))}

if (se1==2){

runclust$taxa<-runclust$taxa%>%

mutate(cluster4=ifelse(cluster4==2,"fi",cluster4))}

if (se1==3){

runclust$taxa<-runclust$taxa%>%

mutate(cluster4=ifelse(cluster4==3,"fi",cluster4))}

#maybe skip this part?

se2<-runclust$taxa%>%

filter(taxon_name=="Sphingomonas 9")

se2<-se2$cluster4

clusseed<-append(clusseed,se2=="fi")

if (se2==1){

runclust$taxa<-runclust$taxa%>%

mutate(cluster4=ifelse(cluster4==1,"se",cluster4))}

if (se2==2){

runclust$taxa<-runclust$taxa%>%

mutate(cluster4=ifelse(cluster4==2,"se",cluster4))}

if (se2==3){

runclust$taxa<-runclust$taxa%>%

mutate(cluster4=ifelse(cluster4==3,"se",cluster4))}

ln<-c(1,2,3)

leftover_no<-ln[ln %!in% c(se1,se2)]

runclust$taxa<-runclust$taxa%>%

mutate(cluster4=ifelse(cluster4==leftover_no[1],"th",cluster4))

t<-runclust$taxa%>%

select(taxon_name,cluster4)

colnames(t)[2]<-c(paste("seed",(1100+i),sep=""))

trunktax<-left_join(trunktax,t)

}

#View(optclust)

hist(optclust, breaks=60)

TRUE %in% clusseed

lt<-trunktax%>%

gather("seed","cluster",-taxon_name,-total_rel_abundance)%>%

replace_na(list(cluster="none"))%>%

group_by(taxon_name)%>%

mutate(pct_1=mean(cluster=="fi"))%>%

mutate(pct_2=mean(cluster=="se"))%>%

mutate(pct_3=mean(cluster=="th"))%>%

select(-seed,-cluster)%>%

unique()%>%

mutate(cluster4=0)

for (row in 1:nrow(lt)) {

gen <- lt[row, "pct_1"]

mid <- lt[row, "pct_2"]

lat <- lt[row, "pct_3"]

if(gen > mid & gen > lat) {

lt[row,"cluster4"]<-1

lt[row,"support"]<-gen

}

if(mid >= gen & mid > lat & mid!=0 ) {

lt[row,"cluster4"]<-2

lt[row,"support"]<-mid

}

if(lat >= gen & lat >= mid & lat!=0 ) {

lt[row,"cluster4"]<-3

lt[row,"support"]<-lat

}

}

taxclu<-lt%>%

select(taxon_name,cluster4)

runcluster<-runl%>%

select_samples(sample_id,type,run, timepoint, days_since_leaf_emergence, tree_id,environment)%>%

add_rel_abundance()

runcluster$taxa<-left_join(runcluster$taxa,taxclu)%>%

filter(cluster4>0)%>%

drop_na(cluster4)

alldata<-left_join(runcluster$abundances,runcluster$taxa)%>%

drop_na(cluster4)

c4data<-alldata%>%

group_by(sample_id,cluster4)%>%

summarize(cluster_abundance = sum(rel_abundance))

sample_id<-rep(runcluster$samples$sample_id,3)

l<-length(sample_id)

cluster4<-c(rep(1,l/3),rep(2,l/3),rep(3,l/3))

complete<-as_tibble(cbind(sample_id,cluster4))

complete$cluster4<- as.integer(complete$cluster4)

c4data<-left_join(complete,c4data, by = c("cluster4" = "cluster4", "sample_id" = "sample_id"))

c4data<-c4data%>%

replace_na(list(cluster_abundance=0))%>%

left_join(runcluster$samples)

c4data$tree_id<-as.character(c4data$tree_id)

ggplot(c4data,aes(x=days_since_leaf_emergence,y=cluster_abundance))+

geom_boxplot(width=3.5,alpha=0.5,color='grey',aes(group=interaction(cluster4, days_since_leaf_emergence)))+

geom_point(size=2,alpha=0.8, aes(shape=environment,color=tree_id))+

#geom_text(label=c4data$tree_id,size=2,vjust=1.5)+

scale_color_manual(values=c("#153940","#8A8A3A","#577A40","#C49443"),guide=FALSE)+

facet_wrap(~cluster4,ncol=3)+

ylab("Relative abundance of cluster")+

xlab("Days since leaf emergence")+

scale_y_continuous(limits=c(0,1.01))+

labs(shape="Land use")+

theme_bw()

ggplot(c4data,aes(x=tree_id,y=cluster_abundance))+

geom_boxplot(alpha=0.5,color='grey',aes(group=interaction(tree_id, cluster4)))+

geom_point(size=2,alpha=0.8, aes(shape=environment,color=tree_id))+

#geom_text(label=c4data$tree_id,size=2,vjust=1.5)+

scale_color_manual(values=c("#153940","#8A8A3A","#577A40","#C49443"),guide=FALSE)+

facet_wrap(~cluster4,ncol=4)+

ylab("Relative abundance of cluster")+

xlab("Tree individual")+

scale_y_continuous(limits=c(0,1.01))+

labs(shape="Land use")+

theme_bw()

c4datap<-c4data%>%

#filter(environment=="park")%>%

filter(cluster4==2)

cor.test(c4datap$cluster_abundance,c4datap$days_since_leaf_emergence,method="kendall")

```

```{r,fig.height = 5}

rm(eartax,gentax,midtax,lattax,on,tw,th,fo,fi,mid,lat,gen,branchtax,leaftax,trunktax,runclust,runclust_scaled,runcluster,runclu,table,clu,clus,c4data,c4datap,clustable,complete,n_clust,row,optclust,seedlate,sphingo,st2,se1,se2,clusseed,cluster4,max_cluster,sample_id,lt,alldata,runl,runr,runf,t,tab,i,l,leftover_no,ln,r,firtax,sectax,taxclu)

```

### Overlap compartments

```{r,fig.height = 5}

taxclust<-taxclust%>%

filter(cluster4!=0)

runc<-run%>%

filter_samples(run=="Antwerp")#%>%

#filter_samples(lib_size>911)%>%

#rarefy(912)

runc$taxa<-left_join(runc$taxa,taxclust)

runl<-runc%>%

filter_samples(type=="Leaves")%>%

add_total_rel_abundance()%>%

add_rel_abundance()%>%

filter_abundances(abundance>10)

runb<-runc%>%

filter_samples(type=="Branch")%>%

add_total_rel_abundance()%>%

add_rel_abundance()%>%

filter_abundances(abundance>10)

runt<-runc%>%

filter_samples(type=="Trunk")%>%

add_total_rel_abundance()%>%

add_rel_abundance()%>%

filter_abundances(abundance>10)

runbd<-runc%>%

filter_samples(type=="Buds")%>%

add_total_rel_abundance()%>%

add_rel_abundance()%>%

filter_abundances(abundance>10)

```

# Contribution to cluster graph: Figure 4A,B

```{r,fig.height = 5}

clustp<-matrix(nrow=0,ncol=6) # proportions

clusta<-matrix(nrow=0,ncol=6) # abundances

ll<-length(taxclust$taxon_name)

for (i in c(1,4,2,3)){ # cluster by cluster

prop<-c()

abun<-c()

taxc<-taxclust%>%

filter(cluster4==i)

time<-(c(11,11,11,11)[i]) # timepoint before which we want to check other niches

time2<-(if (i == 1){seq(1,10,1)}else{

if (i== 2){c(7,8,9)}else{

if (i == 3){c(7,8,9,10)}else{

if(i == 4){c(1,2)}}}}) # time frame in which cluster is relevant

rt<-runt%>%

filter_samples(timepoint<time)

rb<-runb%>%

filter_samples(timepoint<time)

rbd<-runbd

rl<-runl%>%

filter_samples(timepoint %in% time2)

l<- taxc$taxon_name

t<- rt$taxa$taxon_name

b<- rb$taxa$taxon_name

d<- rbd$taxa$taxon_name

lt<-intersect(l,t)

ld<-intersect(l,d)

lb<-intersect(l,b)

all<-union(union(lt,lb),ld)

to<-setdiff(setdiff(lt,b),d)

bo<-setdiff(setdiff(lb,d),t)

do<-setdiff(setdiff(ld,b),t)

inter<-setdiff(setdiff(setdiff(all,bo),to),do)

prop[1]<-c(1,3,4,2)[i] # for ASV proportions

prop[2]<-length(l)/ll

prop[3]<-length(bo)/ll

prop[4]<-length(to)/ll

prop[5]<-length(do)/ll

prop[6]<-length(inter)/ll

abun[1]<-c(1,3,4,2)[i] # for abundance contribution

rlo<-rl

rlo$taxa<-rlo$taxa%>%

filter(taxon_name %in% l)

rlo<-process_taxon_selection(rlo)

abun[2]<-sum(rlo$abundances$rel_abundance)/nrow(rlo$samples)

rbo<-rl

rbo$taxa<-rbo$taxa%>%

filter(taxon_name %in% bo)

rbo<-process_taxon_selection(rbo)

abun[3]<-sum(rbo$abundances$rel_abundance)/nrow(rbo$samples)

rto<-rl

rto$taxa<-rto$taxa%>%

filter(taxon_name %in% to)

rto<-process_taxon_selection(rto)

abun[4]<-sum(rto$abundances$rel_abundance)/nrow(rto$samples)

rdo<-rl

rdo$taxa<-rdo$taxa%>%

filter(taxon_name %in% do)

rdo<-process_taxon_selection(rdo)

abun[5]<-sum(rdo$abundances$rel_abundance)/nrow(rdo$samples)

rio<-rl

rio$taxa<-rio$taxa%>%

filter(taxon_name %in% inter)

rio<-process_taxon_selection(rio)

abun[6]<-sum(rio$abundances$rel_abundance)/nrow(rio$samples)

clustp<-rbind(clustp,prop)

clusta<-rbind(clusta,abun)

}

colnames(clusta)<-c("Cluster","Leafcluster","Branch","Trunk","Buds","Combination")

colnames(clustp)<-c("Cluster","Leafcluster","Branch","Trunk","Buds","Combination")

clusta1<-clusta%>%

as.tibble()%>%

select(Cluster,Leafcluster)%>%

gather(-Cluster, key = "Niche", value = "ASVabundance")

clusta2<-clusta%>%

as.tibble()%>%

select(-Leafcluster)%>%

gather(-Cluster, key = "Niche", value = "ASVabundance")

clustp1<-clustp%>%

as.tibble()%>%

select(Cluster,Leafcluster)%>%

gather(-Cluster, key = "Niche", value = "ASVproportion")

clustp2<-clustp%>%

as.tibble()%>%

select(-Leafcluster)%>%

gather(-Cluster, key = "Niche", value = "ASVproportion")

clusta1$ASVabundance<-as.numeric(as.character(clusta1$ASVabundance))

clusta2$ASVabundance<-as.numeric(as.character(clusta2$ASVabundance))

clustp1$ASVproportion<-as.numeric(as.character(clustp1$ASVproportion))

clustp2$ASVproportion<-as.numeric(as.character(clustp2$ASVproportion))

clusta2$Niche <- ordered(clusta2$Niche , levels = c("Cluster","Combination","Buds","Branch","Trunk"))

clustp2$Niche <- ordered(clustp2$Niche , levels = c("Cluster","Combination","Buds","Branch","Trunk"))

barwidth = 0.50

View(clustp2)

View(clustp1)

sum(0.016574586,0.055248619,0.005524862,0.497237569)/0.63535912 # cluster 1, general

sum(0.005524862,0.027624309,0.011049724)/0.09392265 # cluster 3, mid

sum(0.060773481,0.038674033)/0.19889503 # cluster 4, late

View(clusta2)

View(clusta1)

sum(0.002217687,0.047670404,0.001531729)/0.08872628 #mid

sum(0.076312265,0.085319659)/0.24893655 #late

ggplot() +

geom_bar(data=clustp1, aes(x=Cluster,y=ASVproportion, fill=Niche),stat="identity", position=position_dodge(), width = barwidth)+

geom_bar(data=clustp2, aes(x=(Cluster+(barwidth/2)) ,y=ASVproportion, fill=Niche),stat="identity", position="stack", width = barwidth)+

xlab("")+

geom_text(size=3,aes(x=1+barwidth/4, y=-0.02, label="General"))+

geom_text(size=3,aes(x=2+barwidth/4, y=-0.02, label="Early"))+

geom_text(size=3,aes(x=3+barwidth/4, y=-0.02, label="Mid"))+

geom_text(size=3,aes(x=4+barwidth/4, y=-0.02, label="Late"))+

scale_fill_manual(values=c("darkorange3","darkgreen","firebrick3","chartreuse3","darkorange4"))+

#scale_y_continuous(limits=c(-0.01,0.12))+

ylab("Proportion of leaf ASVs")+

labs(fill = "Compartment")+

theme_bw()+

theme(axis.text.x = element_blank(),

axis.ticks.x=element_blank(),

panel.grid.major = element_blank(),

panel.grid.minor = element_blank())

ggplot() +

geom_bar(data=clusta1, aes(x=Cluster,y=ASVabundance, fill=Niche),stat="identity", position=position_dodge(), width = barwidth)+

geom_bar(data=clusta2, aes(x=(Cluster+(barwidth/2)) ,y=ASVabundance, fill=Niche),stat="identity", position="stack", width = barwidth)+

xlab("")+

geom_text(size=3,aes(x=1+barwidth/4, y=-0.02, label="General"))+

geom_text(size=3,aes(x=2+barwidth/4, y=-0.02, label="Early"))+

geom_text(size=3,aes(x=3+barwidth/4, y=-0.02, label="Mid"))+

geom_text(size=3,aes(x=4+barwidth/4, y=-0.02, label="Late"))+

scale_fill_manual(values=c("darkorange3","darkgreen","firebrick3","chartreuse3","darkorange4"))+

labs(fill = "Compartment")+

ylab("Relative abundances on leaves")+

theme_bw()+

theme(axis.text.x = element_blank(),

axis.ticks.x=element_blank(),

panel.grid.major = element_blank(),

panel.grid.minor = element_blank())

```

# Generalism in compartments and ubiquity in trees

```{r,fig.height = 5}

runf<-run%>%

filter_samples(run=="Antwerp")%>% # or skip this filter for including Milan

#filter_samples(run=="Milan")%>%

add_rel_abundance()%>%

filter_abundances(abundance>10)

# taxon name with ubiquity

ubi<-everything(runf)%>%

group_by(taxon_name)%>%

summarize( Ubiquity = n_distinct(tree_id))%>%

drop_na()

# taxon name with ubiquity with cluster

taxclust<-taxclust%>%

filter(cluster4>0)

generalists<-ubi%>%

left_join(taxclust)%>%

drop_na()%>%

mutate(cluster4=ifelse(cluster4==4,"Early",cluster4))%>%

mutate(cluster4=ifelse(cluster4==3,"Late" ,cluster4))%>%

mutate(cluster4=ifelse(cluster4==2,"Mid",cluster4))%>%

mutate(cluster4=ifelse(cluster4==1,"General",cluster4))

generalists$Ubiquity<-as.numeric(generalists$Ubiquity)

# numbers per cluster and ubiquity

gts<-generalists%>%

group_by(cluster4,Ubiquity)%>%

summarize(count=n())%>%

mutate(taxa=sum(count))%>%

mutate(perc=count/taxa)

gts$cluster4 <- ordered(gts$cluster4 , levels = c("General","Early","Mid","Late"))

```

# Figure 5C and D

```{r,fig.height = 5}

cols<-c( "#e0ce6d", "#d49727", "#aa601b", "#3c140a")

cols<-c( "#e0ce6d", "#ddad38", "#d49727", "#cb8117", "#aa601b", "#87431a", "#612a15", "#3c140a")

gts$Ubiquity<-as.factor(gts$Ubiquity)

barwidth<-0.5

ggplot(gts,aes(x=cluster4,y=perc))+

geom_bar(stat="identity",aes(fill=Ubiquity), width = barwidth)+

xlab("")+

ylab("Proportion of ASVs")+

scale_fill_manual(values = cols,name="Ubiquity\n(# trees)")+ #Antwerp\n

theme_bw()+

theme(panel.grid.major = element_blank(), panel.grid.minor = element_blank())

```

```{r,fig.height = 5}

rm(lb,ld,ll,lt,bo,d,do,inter,prop,time,time2,to,rbo,rdo,rio,rl,rlo,rt,rto,rb,rbd,clusta,clusta1,clusta2,clustp,clustp1,clustp2,t,l,i,cols,barwidth,b,all,abun,ubi,taxc,runt,runl,runf,runc,runbd,runb,generalists,gts)

```
